# The Vertebrate *RCAN* Gene Family: Novel Insights into Evolution, Structure and Regulation

**DOI:** 10.1371/journal.pone.0085539

**Published:** 2014-01-20

**Authors:** Eva Serrano-Candelas, Domènec Farré, Álvaro Aranguren-Ibáñez, Sergio Martínez-Høyer, Mercè Pérez-Riba

**Affiliations:** 1 Cancer and Human Molecular Genetics Department, Bellvitge Biomedical Research Institute – IDIBELL, L’Hospitalet de Llobregat, Barcelona, Spain; 2 Biological Aggression and Response Mechanisms Unit, Institut d'Investigacions Biomèdiques August Pi i Sunyer – IDIBAPS, Barcelona, Spain; VIB & Katholieke Universiteit Leuven, Belgium

## Abstract

Recently there has been much interest in the Regulators of Calcineurin (RCAN) proteins which are important endogenous modulators of the calcineurin-NFATc signalling pathway. They have been shown to have a crucial role in cellular programmes such as the immune response, muscle fibre remodelling and memory, but also in pathological processes such as cardiac hypertrophy and neurodegenerative diseases. In vertebrates, the RCAN family form a functional subfamily of three members RCAN1, RCAN2 and RCAN3 whereas only one RCAN is present in the rest of Eukarya. In addition, *RCAN* genes have been shown to collocate with *RUNX* and *CLIC* genes in ACD clusters (ACD21, ACD6 and ACD1). How the *RCAN* genes and their clustering in ACDs evolved is still unknown. After analysing *RCAN* gene family evolution using bioinformatic tools, we propose that the three *RCAN* vertebrate genes within the ACD clusters, which evolved from single copy genes present in invertebrates and lower eukaryotes, are the result of two rounds of whole genome duplication, followed by a segmental duplication. This evolutionary scenario involves the loss or gain of some *RCAN* genes during evolution. In addition, we have analysed *RCAN* gene structure and identified the existence of several characteristic features that can be involved in *RCAN* evolution and gene expression regulation. These included: several transposable elements, CpG islands in the 5′ region of the genes, the existence of antisense transcripts (NAT) associated with the three human genes, and considerable evidence for bidirectional promoters that regulate *RCAN* gene expression. Furthermore, we show that the CpG island associated with the *RCAN3* gene promoter is unmethylated and transcriptionally active. All these results provide timely new insights into the molecular mechanisms underlying RCAN function and a more in depth knowledge of this gene family whose members are obvious candidates for the development of future therapies.

## Introduction

The Regulators of Calcineurin proteins (RCAN, formerly known as DSCR and calcipressin, amongst other terms) are important regulators of several cellular programmes [1]. RCAN proteins are also involved in the development of several pathological conditions such as Down’s syndrome, cardiac hypertrophy and Alzheimer's disease, amongst others [2]–[6]. At the mechanistic level, RCANs have been mainly described to act through physical binding and modulation of the Ca^2+^ and calmodulin-dependent serine-threonine phosphatase calcineurin (Cn; also known as PPP3, formerly PP2B) [1], [7]–[9]. This enzyme has many important physiological substrates including the transcription factors cytosolic Nuclear Factors of Activated T cells (NFATc) [10]. Activated Cn dephosphorylates their substrates, the NFATc proteins, which then translocate to nuclei, where they induce NFATc-mediated gene expression in many cell types. The Cn-NFATc signalling pathway is a crucial regulator of several biological processes such as: lymphocyte activation, angiogenesis, morphogenesis of the heart valves and neural and muscle development (reviewed in [11]). It is worth noting that Cn is present in all the Eukarya and that the NFATc proteins are restricted to vertebrates (reviewed in [10]).

Among the endogenous inhibitors of Cn, the RCAN proteins bind to Cn and in this way modulate Cn-NFATc signalling in mammals. In this context, RCANs have been described as being able to facilitate or inhibit Cn-NFATc signalling, depending on the RCAN protein levels and the affinity for Cn of different RCAN binding sites [12]–[15].

In almost all jawed vertebrates there are three members of the *RCAN* gene family: *RCAN1*, *RCAN2* and *RCAN3*, each of them coding for several transcripts and protein isoforms (reviewed in [1]) whereas only one member is found in most invertebrates, fungi and protozoa [16]. In jawed vertebrates, RCAN proteins share a high amino acid sequence identity in the central and C-terminal regions but have different amino-terminal regions. In the common region, the FLISPP motif, highly conserved in all the Eukarya, has until now been considered to be the signature of this family. Recently, additional conserved motifs, which are encoded by the last two exons of RCANs, have been described [14]. They include the PXIXIT and LXXP motifs, present in all eukaryotic organisms that bind to Cn and modulate Cn-NFATc signalling. Due to the multiple *RCAN* genes in vertebrates and the high amino acid identity of the central and C-terminal regions of the three RCAN proteins, together with their conserved regulatory function towards Cn-NFATc signalling, these proteins constitute a functional subfamily among the eukaryotic RCAN family in jawed vertebrates [16].

The phylum chordata can be subdivided into four superclasses, that emerged subsequently: Urochordata, Cephalocordata, (both invertebrates), Agnatha (jawless vertebrates that can be subdivided in two subclasses: Hyperotreti or Myxini (hagfishes) and Hyperoartia (lampreys)) and Gnathostomata (jawed vertebrates). It is believed that the original invertebrate genomes suffered two rounds of whole genome duplication (1R-WGD and 2R-WGD) that gave rise to the emergence of gnathostomes: the 2R hypothesis (proposed by [17] and reviewed in [18]). This hypothesis implies the existence of four vertebrate orthologs for each gene in invertebrates, known as the “one-to-four rule”. Since not all genes fit within this rule it is assumed that gene deletion or amplification has taken place in order to fit the 2R hypothesis. It has been considered that the divergence between agnathans and gnathostomes occurred at some time before the 2R-WGD [19]–[21], however, recent studies suggest that at least the sea lamprey genome has also suffered this second whole genome duplication event [22]–[24].

Vertebrate *RCAN* genes have been described as mapping within ACD clusters (for *AML* (later renamed as *RUNX*), *CLIC* and *DSCR* (renamed as *RCAN*) genes) [25]. Specifically, human *RCAN1* (*hRCAN1*) is located in the chromosome 21 ACD21 cluster, human *RCAN2* (*hRCAN2*) in the chromosome 6 ACD6 cluster and human *RCAN3* (*hRCAN3*) in the chromosome 1 ACD1 cluster. It has been previously postulated that the three genes have evolved from successive gene duplications during the two rounds of WGD [25].

Regarding the *RCAN* gene family, little is known about their general characteristic structural traits and gene expression regulation. It has been shown that *RCAN1-1* transcription is mainly regulated by glucocorticoids and *RCAN1-4* is transcriptionally activated by the calcium dependent NFATc and C/EBPβ transcription factors, osmotic and oxidative stress and steroid hormones, amongst others, whereas *RCAN2-4* is regulated by thyroid hormone [1], [26]. Regarding the *RCAN3* gene, there are no functional studies of its gene expression regulation. Recently, it has been described that *hRCAN3* gives rise to 21 different possible transcripts based on RT-PCR analysis [27]–[31]. In addition, this gene bears a putative bidirectional promoter that might control the expression of four different *RCAN3* natural antisense transcripts (NATs), called *RCAN3AS*, that are formed by combinations of three different exons, the first and the third being common to all of them [31]. However, neither protein detection nor functional effect has been reported up to now for these NATs.

Here, for the first time we analyse the evolution of the three *RCAN* genes present in almost all jawed vertebrates, describe the structural conservation of human *RCAN* genes and suggest the existence of several associated NATs, which include some conserved transposon sequences in all *RCAN* genes. In addition, all three *RCAN* gene promoter regions include CpG islands and we can also conclude that at least the *RCAN3*-associated CpG island is functional.

## Materials and Methods

### Genomic Sequences Retrieval and Nomenclature

Ensembl database (http://www.ensembl.org and http://metazoa.ensembl.org; release 73, September 2013) [32], Biomart tool implemented in Ensembl (www.biomart.org) [33], UCSC database (http://genome.ucsc.edu/) [34], NCBI Reference Sequence (RefSeq) database (http://www.ncbi.nlm.nih.gov/RefSeq/) [35], and BLAST (http://blast.ncbi.nlm.nih.gov/) [36] were used for sequence searching and retrieval of the different genomic and protein sequences.

The following nomenclature has been used: italicized letters for genes and transcripts and non-italicized for proteins; all letters in uppercase for human and primate genes and proteins, and first letter only in upper case (with the remaining in lower case) for all other organisms with the exception of zebrafish and Sauria, for which the convention in the field is to use all lower case. To refer to the gene and protein family in general, all letters in uppercase were used.

### Sequence Alignments and Analysis

Alignments of human RCAN proteins and *RCAN3*-associated CpG islands were performed using MAFFT v.6 online version (http://mafft.cbrc.jp/alignment/software/) [37] with the following parameters: FFT-NS-I method, scoring matrix BLOSUM62, Gap opening penalty = 3, and offset value = 1. These alignments were subsequently edited using GeneDoc software (http://www.nrbsc.org/gfx/genedoc/) [38]. Alignments and pip-type graphs of *RCAN*-associated antisense transcripts were achieved using multi-zPicture (http://zpicture.dcode.org/multiz.php) [39] applying an ECR criteria of ≥10 pb; ≥50% ID. Transposable element sequences were identified using the CENSOR tool (http://www.girinst.org/censor/index.php) [40]. Transcription factor binding sites were predicted using PROMO software v.3 (http://alggen.lsi.upc.es/cgi-bin/promo_v3/promo/promoinit.cgi?dirDB=TF_8.3) [41], [42] selecting *Homo sapiens* or eutherian weight matrices, a maximum matrix dissimilarity rate = 5%, and binding site length ≥6 nt.

### Evolutionary Trees

Evolutionary analyses were conducted in MEGA5 [43]. Alignments used for phylogenetic analysis were performed by Muscle software (http://www.ebi.ac.uk/Tools/msa/muscle/) [44] implemented in MEGA5 with the following parameters: UPGMB clustering method, Gap opening penalty = −400, and iterations = 10. All positions with less than 75% site coverage were eliminated for the analysis. That is to say, fewer than 25% alignment gaps, missing data, and ambiguous bases were allowed at any position. Phylogenic trees and evolutionary distances were inferred by using the Maximum Likelihood method based on the Hasegawa-Kishino-Yano model [45]. The percentage of replicate trees in which the associated taxa clustered together in the bootstrap test (1000 replicates) is shown next to the branches [46]. Initial tree(s) for the heuristic search were obtained automatically by applying Neighbor-Join and BioNJ algorithms [47], [48] to a matrix of pairwise distances estimated using the Maximum Composite Likelihood (MCL) approach [49], and then selecting the topology with superior log likelihood value. A discrete Gamma distribution (+G) with 5 categories was used to model evolutionary rate differences among sites. Trees are drawn to scale, with branch lengths in the units of the number of base substitutions per site.

### Searching for Paralogous Genes

Ideograms of chromosome 1 and 6 at 850-band resolution level were obtained from NCBI Genome Decoration Page (GDP) (http://www.ncbi.nlm.nih.gov/genome/tools/gdp/; GRCh37/hg18) [50]. They were used to locate the chromosome 1 and 6 duplicated segment by adding RefSeq genes locations as a custom track retrieved from UCSC Table Browser utility (http://genome.ucsc.edu/cgi-bin/hgTables) [34] as gene transfer format (GTF). Paralogous genes were identified using “Paralogons in the Human Genome” v.5.28 database (http://wolfe.gen.tcd.ie/dup/) [51], as a preliminary approach and subsequently corroborated as current functional paralogous and complemented with data from Ensembl (http://www.ensembl.org) [32] using GRCh37/hg19 genome version assembly. The identified paralogs were manually represented in a non-scaled bar.

### Comparative Analysis of Genome Sequences

The VISTA tool (http://pipeline.lbl.gov) [52] implemented in UCSC genome browser (http://genome.ucsc.edu) [34] was used for multiple alignment to compare with the human sequence. Two versions of the human genome were used to perform the multiple comparative genome analysis: hg19 (GCRh37), last human genome version, was used for alignments with mouse (Shuffle-LAGAN (SLAGAN) alignment version) and primate sequences, while, due to incompleteness of hg19 version alignment data, hg18 (NCBI36) human genome version was used for alignments with mouse sequence (PROLAGAN alignment version) and sequences of the other vertebrates studied. All alignments are PROLAGAN alignments except when otherwise specified. The vertebrate genomes used in the alignments against human sequences were: *Pan troglodites* (panTro, Chimpanzee; Mar 2006), *Pongo abelii* (ponAbe, Orangutan; Jul 2007), *Gorilla gorilla* (gorGor; Dec 2009; SLAGAN alignment), *Callithrix jacchus* (calJac, Marmoset; Jun 2007), *Macaca mulatta* (rheMac; Jan 2006), *Mus musculus* (musMus/S; Jul 2007; SLAGAN alignment), *Equus caballus* (equCab, Horse; Jan 2007), *Canis lupus familiaris* (canFam, Dog; May 2005 v.80), *Mus musculus* (musMus/P; Jul 2007), *Rattus norvegicus* (ratNor, Rat; Nov 2004), *Gallus gallus* (galGal, Chicken; May 2006 v.55), *Danio rerio* (danRer, Zebrafish; Mar 2006; SLAGAN alignment), and *Xenopus tropicalis* (xenTro, Frog; Aug 2005; SLAGAN alignment).

### Luciferase Reporter Gene Assay

Constructs were obtained by PCR with specific primers (−4775Kb_TSS_hRCAN3_Fw (CCAACTGATCCACCCACCTTGG); −1999Kb_Fw (CCACTTGTATCATTTTCATA); 699pb_Fw (ATCTCATTTGATGTGAAAACTC); −281pb_Fw (GGAGTAAGAGGAGGAGGGAG); +550pb_Rv (CGCCAGAGGTCCTGTTTTC)), using the BAC clone RP4-633K13 obtained from the Children's Hospital Oakland Research Institute (CHORI) (http://www.chori.org/) as a template and then cloning into pGL3-luc Basic reporter vector (Promega, Madison, USA). All DNA sequences were confirmed by DNA sequencing. HEK 293T cells were seeded at 50000 cells/well in 24-well plates. 24 h later, each well was transfected with 30 fmol of each construct and 1 ng of pRLNull vector (Promega) as an internal transfection control. Empty pGL3 vector was included in the analysis as control. The total amount of plasmid DNA was kept constant in every condition using empty pCDNA3.1 vector (Invitrogen Corporation, Carlsbad, USA). 48 h after transfection, cells were lysed and analyzed using the Dual Luciferase Reporter Assay (Promega) following the manufacturer’s protocol on a multiplate luminometer (FLUOstar Optima, BMG). Luciferase units were normalized to Renilla luciferase values.

## Results

### Overall Evolution of *RCAN* Genes

**Figure 1:**
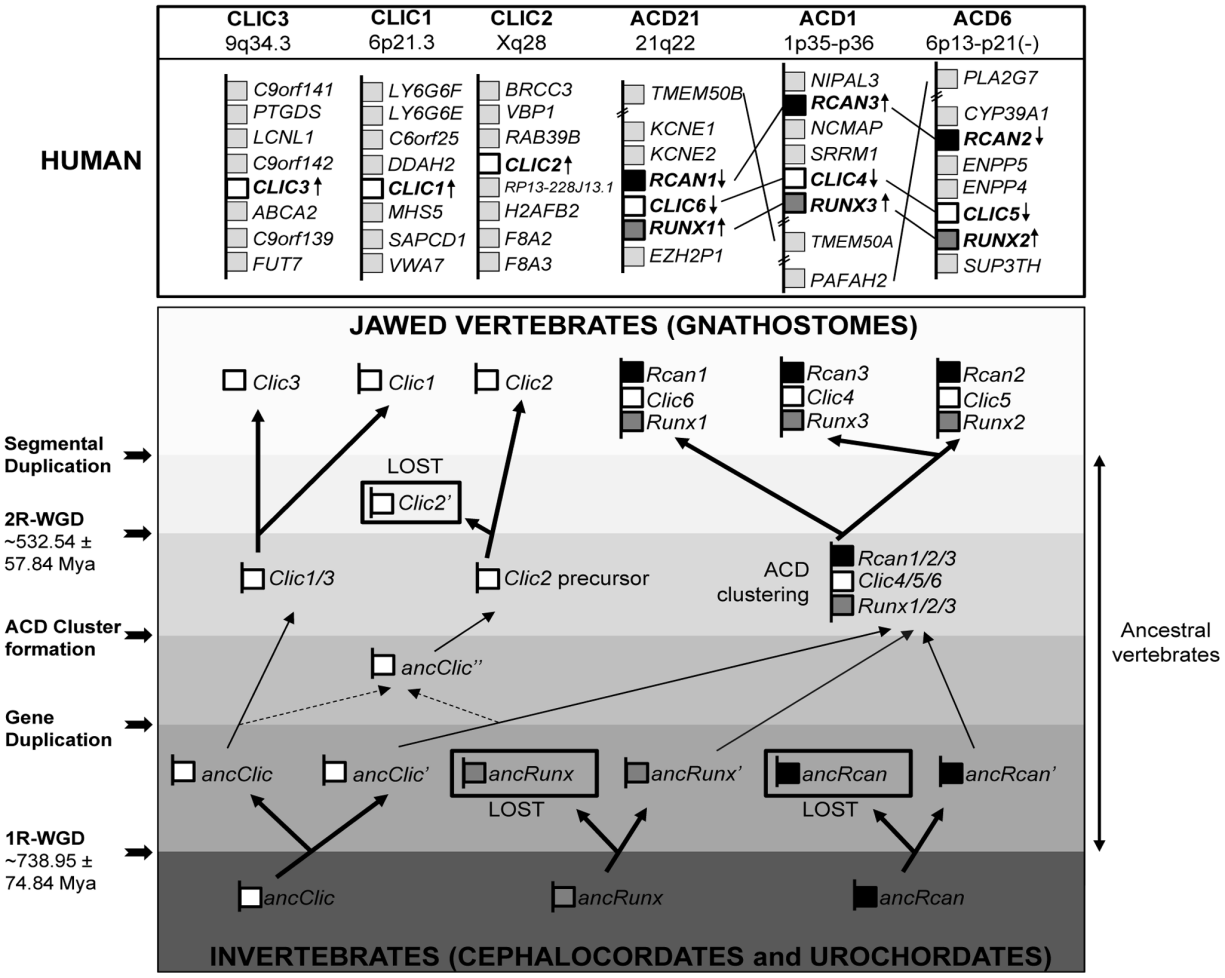
RCAN/CLIC/RUNX (ACD) clustered genes evolution in gnasthostomes. Invertebrates have a single copy of *Rcan*, *Clic* and *Runx* genes, while in jawed vertebrates they diverged into multigenic families. In jawed vertebrates, all members of *Runx* and *Rcan* families and three members of the *Clic* family are located in ACD clusters. The two rounds of whole-genome duplication (1R-WGD and 2R-WGD) occasioned the appearance of *Clic3*, *Clic1*, ACD21 cluster, and ACD1/6 cluster precursor. A posterior segmental duplication may be the origin of the ACD1 and ACD6 clusters. *Clic2* ancestral precursor (ancClic'') may have originated from gene duplication, before the 2R-WGD from either *Clic1/3* or *Clic4/5/6* precursor (dashed lines). Thicker arrows indicate gene duplication events and gene losses are shown framed in black. Estimated times of 1R-WGD and 2R-WGD were obtained from *Vienne et al.* [58]. Human genes, their chromosomal context and their chromosome locations are indicated in the upper panel (HUMAN). *RCAN, CLIC* and *RUNX* genes are represented in black, white and dark grey boxes, respectively, while other human genes are represented in light grey. The ACD6 cluster is represented in the opposite direction (−). Double transversal lines indicate that some genes were omitted in the representation for simplicity, arrows indicate the direction of the gene transcription and connecting lines link paralogous genes. Abbreviations: anc, ancestral; agn, agnathans; gna, gnathostomes; Mya, Million Years Ago; WGD, Whole Genome Duplication.

We have previously reported that the *RCAN* family consists of three genes that constitute a functional subfamily in gnathostomes, with the exception of some fishes (*Tetraodon nigroviridis* (tetraodon) and *Takifugu rubripens* (fugu)), while only one *RCAN* gene is found in the rest of the Eukarya [16]. In jawed vertebrates (from here on referred to as vertebrates unless otherwise specified), Strippoli and colleagues mapped *RCAN* genes within the ACD clusters, together with *RUNX* and *CLIC* genes [25] (Figure 1). In a similar way to the *RCAN* gene family, the *RUNX* and *CLIC* vertebrate gene families also include several genes. In spite of some particular exceptions, there are six *CLIC* genes (*CLIC1-6*) and three *RCAN* and *RUNX* genes (*RUNX1-3*) in vertebrates [25]. Based on these previous findings we decided to further investigate the evolution of these ACD clustered genes by performing an exhaustive search for *RUNX*, *CLIC* and *RCAN* orthologs in Chordata organisms in public databases such as Ensembl [32], RefSeq [35] and UCSC [34], and the BLAST alignment tool [36].

The analysis of *RUNX*, *CLIC* and *RCAN* genes in invertebrate chordates shows the presence of one human orthologous gene for each of them in *Ciona intestinalis* (sea squirt), used as representative specie of Urochordata (Figure 1, invertebrates). Additionally, they are located in different genomic locations. The ortholog of the human *RCAN* (*CiRcan*; *ENSCING00000013221*) maps at chromosome 8 and both *RUNX* (*CiRunx*; *ENSCING00000002253*) and *CLIC* (*CiClic*; *ENSCING00000004649* (*CIN.26129*)) orthologs map at chromosome 12, but they are separated by 1 Mb. In the same context, the search in the RefSeq database [35] carried out for *Branchiostoma floridae* (amphioxus), as the representative of the invertebrate Cephalochordata, identified one *Rcan* (Gene ID: 7217844), one *Runx* (Gene ID: 7214657), and one *Clic* (Gene ID: 7206331) as possible orthologs, all of them located in different scaffolds (this genome is still incompletely assembled). Therefore, these results, mainly from *Ciona intestinalis*, point to *RUNX*, *CLIC* and *RCAN* genes being unique and not clustered in invertebrate chordate organisms (Figure 1, invertebrates).

We also analysed the presence of *RUNX*, *CLIC* and *RCAN* genes in jawless vertebrates (agnathans). It has been previously reported that both *Myxine glutinosa* (Atlantic hagfish) and *Petromyzon marinus* (sea lamprey) (from here on referred to as hagfish and lamprey, respectively, unless otherwise specified) have two copies for *Runx* genes, called *RunxA* and *RunxB* (Figure S1) [53], [54]. In the case of hagfish, they correspond to *DQ990008* and *DQ990009* genes in the RefSeq database [35]. In the Ensembl database [32] the lamprey *ENSPMAG00000000391* gene appears as an ortholog for human *RUNX1* gene, and would constitute the *RunxA* gene of lamprey, while *RunxB* gene has not been yet annotated. By genomic comparison using human *RUNX2* and *RUNX3* (*hRUNX2* and *hRUNX3*) as templates, we localized a partial region of the putative lamprey *RunxB* gene in the scaffold GL476719.

When searching for orthologs for human *RCAN* genes in these organisms, we did not retrieve any result for Atlantic hagfish or sea lamprey. However, when we extended the searching of *RCAN* orthologs in other species of lampreys, by using TBLASTN [55] against whole-genome shotgun contigs (WGS), we were able to find a putative *Rcan* gene in *Lethenteron camtschaticum* (Arctic lamprey), the genome of which has been recently sequenced (NCBI Bioproject PRJNA192554). This result suggests that *RCAN* genes actually exist in agnathans.

Concerning *CLIC* genes, we were not able to find any orthologous gene in Atlantic hagfish, whereas three *CLIC* genes were found in lamprey: *Clic1*: *ENSPMAG00000002107*, *Clic5*: *ENSPMAG00000000089* and *Clic6: ENSPMAG00000002003*, an incomplete annotated sequence (Figure S1). In order to have a general view of the relationship of lamprey *Clic* genes with *CLIC* genes of jawed vertebrates, we performed a refined phylogenetic analysis for all *CLIC* genes except for the incomplete *LpClic6* sequence (Figure S2). Our analysis is consistent with the phylogenetic tree published in the Ensembl database (ENSGT00550000074477) [32] and suggests that *LpClic1* and *LpClic5* genes diverged in the very early stages of vertebrate evolution. Regarding *LpClic6* present in lamprey, the “supporting evidence” section of the Ensembl database [32] and our DELTA-BLAST [56] search suggest that its protein product is related to human CLIC2 protein. However, the orthologs of *LpClic6* annotated in the Ensembl database are human *CLIC4*, *CLIC5*, and *CLIC6* and the phylogenetic tree from Ensembl (ENSGT00550000074477) [32] relates this sequence with *hCLIC6*. Detailed analysis revealed that the annotation for *LpClic6* sequence is incomplete as only two exons are annotated, although there is evidence for the existence of more exons. For this reason, it is difficult to determine the origin of *LpClic6* exactly, but its protein product seems to be related to hCLIC2.

Gnasthostomes, despite some isolated exceptions, have three ACD clusters, corresponding to human ACD21, ACD6 and ACD1 [25] (Figure 1). Additionally, the majority of them have three additional *CLIC* genes (corresponding to human *CLIC1* (Chromosome 6), *CLIC2* (Chromosome X) and *CLIC3* (Chromosome 9)).

The phylogenetic analysis of *CLIC* genes (Figure S2 and ENSGT00550000074477 tree from Ensembl [32]) suggests that there was an initial divergence of the precursor of *Clic1* and *Clic3* genes (*Clic1/3*) from the precursor of *Clic4*, *Clic5* and *Clic6* genes (*Clic4/5/6*). Later, there was a duplication and posterior split of *Clic1/3* precursor and a triplication and split of the *Clic4/5/6* precursor. One plausible explanation for this scenario for *Clic* genes is depicted in Figure 1, which is in line with previously reported vertebrate phylogenetic studies on ACD members, which indicate that the genes of the ACD21 cluster are more ancestral than the members of the ACD6 and ACD1 clusters, which seem to have appeared at the same time later in evolution [16], [25], [57].

Taking all this data into account, we suggest that one of the two copies of the *Runx* and *Rcan* ancestral genes generated after the 1R-WGD were lost, but the same did not happen to the *Clic* genes. Afterwards *Runx*, *Rcan* and one *Clic* came to be clustered together and generated the ancient ACD cluster precursor (*Runx1/2/3-Clic4/5/6-Rcan1/2/3*) (Figure 1). This event would have taken place in the early predecessor of vertebrates, before the 2R-WGD, but after the 1R-WGD, because *Clic*, *Runx* and *Clic* are not clustered in any of the invertebrate chordata organisms analysed. Afterwards, the 2R-WGD, around 532 million years ago (Mya) [58], would have led to the divergence of the current ACD21 cluster (*Runx1-Clic6-Rcan1* genes; in the human genome at 21q22.12) from the ACD1 and ACD6 cluster precursor. At the same time, the split of the current genes *Clic3* (in human genome at 9q34.3) and *Clic1* (in human genome at 6p21.3) (Figure 1) took place.

In order to elucidate a possible origin for *Clic2*, we analysed in detail the phylogenetic tree for *CLIC* genes (Figure S2 and ENSGT00550000074477 from Ensembl [32]). This phylogenetic analysis showed that the *Clic2* genes have more sequence similarity to the *CLIC* genes located within ACD clusters (*Clic4*, *Clic5* and *Clic6*) than to the rest of *CLIC* genes (*Clic1* and *Clic3*). Therefore, we hypothesized that the early precursor of *Clic2* gene (Figure 1, *ancClic’’*) probably resulted from a single gene duplication of the *ancClic’* gene (*Clic4/5/6* precursor gene) generated after the 1R-WGD (Figure 1, dashed lines). However, we cannot rule out that this *ancClic’’* gene could have emerged from gene duplication of the *ancClic* gene (*Clic1/3* precursor) after the 1R-WGD (Figure 1, dashed lines). It is noteworthy that the 2R-WGD event would have generated an additional copy of the *Clic2* gene (Figure 1, *Clic2’*) that must have subsequently disappeared, as it cannot be found in any of the jawed vertebrates analysed.

Regarding the presence of three ACD clusters in almost all gnasthostomes instead of the two expected by the 2R hypothesis, an additional duplication event seems to be required to explain it. We propose that, subsequently to the 2R-WGD, a large-scale segmental duplication and translocation between the two ancestral chromosomes corresponding to human chromosome 1 and 6 would have resulted in the appearance of the current clusters ACD6 (*Runx2-Clic5-Rcan2*; in the human genome at 6p13.3) and ACD1 (*Runx3-Clic4-Rcan3*; in the human genome at 1p35.3) present in almost all jawed vertebrates. This segmental duplication event seems to have already happened in the early jawed vertebrates, considering that the three ACD clusters are already present in *Latimeria chalumnae* (coelacanth), a big marine fish, representative of Sarcopterygii, that split from the rest of the fish more than 400 Mya (between 416 to 450 Mya) [59], [60], and from the rest of the sarcopterygians 410–415 Mya [61].

**Figure 2:**
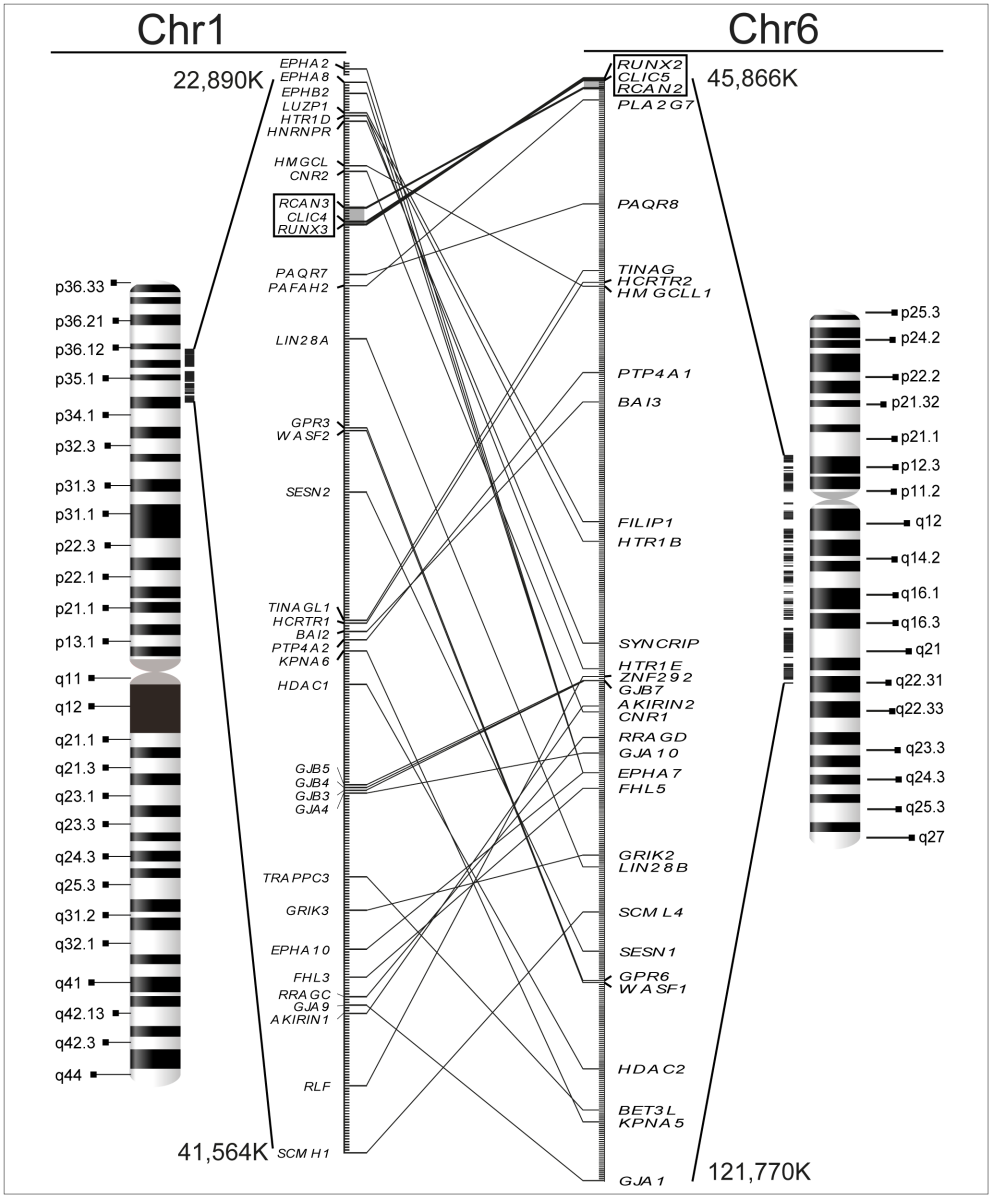
Large-scale segmental duplication between chromosomes 1 and 6 determined by the presence of paralogous genes. The existence of more than 30 paralogous genes located within the flanking regions of the ACD1 and ACD6 clusters (marked in grey) suggests a large-scale (>18 Mb) segmental duplication between chromosome 1 (HSA 1, p-arm) and chromosome 6 (HSA 6, p- and q-arms). Each line connects two paralogous genes. Ideograms of both chromosomes are displayed, where dark and white bands represent G and R bands, respectively. Indicated chromosome coordinates serve as a guide to positioning the duplicated segment.

To reinforce our hypothesis that a segmental duplication event took place between ancestral chromosomes corresponding to human 1 and 6, we decided to look for paralogous genes surrounding these two ACD1 and ACD6 clusters (Figure 2). By using the “Paralogons in the Human Genome v.5.28″ tool [51] to find sets of chromosome regions with a common origin, and manually searching for paralogous genes in the Ensembl database [32] we were able to delimitate a large segment of human chromosome 1 (1p32-p36.3; >18 Mb) that contains functional paralogs in chromosome 6 (6p12-p21.2/q12-q22.1; >75 Mb). Figure 2 shows a view of the duplicated region that includes around 35 pairs of paralogous genes, which are summarized in Table S1. Additionally, we also looked for paralogs of these genes in the lamprey, where we found either none or only a single copy for them, supporting the hypothesis that this segmental duplication did not occur in lamprey. Unfortunately, it was impossible to examine this correctly in the hagfish because its genome has not been completely sequenced yet.

Therefore, our comparative genomic and phylogenetic studies indicate that the ACD clusters present in jawed vertebrates have evolved through two rounds of whole genome duplication and one segmental duplication event. In the case of lamprey, due to the controversy about the lamprey genome and the 2R-WGD, we suggest two different models of gene gain/loss for this organism, depending on whether they have suffered one or two rounds of whole genome duplication (Figure S1). Although we cannot determine which hypothesis is the correct one due to the insufficient genome data available, both models are compatible with our general evolutionary hypothesis for jawed vertebrates.

### Particularities of *RCAN* Genes Evolution in Vertebrates

Regarding the presence of *RCAN* genes in vertebrate organisms, although it has been reported that there are three *RCAN* genes in almost all jawed vertebrates and only one gene in most of the rest of Eukarya, we have found some particular exceptions to this general rule. For *Sorex araneus* (common shrew), *Taeniopygia guttata* (zebra finch), *Procavia capensis* (hyrax) and *Vicugna pacos* (alpaca), no corresponding human *RCAN1* ortholog has been described yet. However, all of them are novel genomic sequence versions that are incompletely assembled and contain numerous sequence gaps. By means of comparative genomic analysis, we have been able to locate and annotate putative *RCAN1* coding sequences for all of them (scaffold 232239, chromosome 1B random, scaffold 13048 and scaffold 2225, respectively).

For *Dario rerio* (zebrafish) one additional *rcan* gene (*ENSDARG00000003109*; named *rcan1a*) has been annotated in the Ensembl database [32]. The origin of this fourth gene can be explained by a recombination of *rcan3* (*ENSDARG00000032623) and rcan1b* (*ENSDARG00000041157*) genes. This hypothesis is supported by the presence of the *srrm1* paralogous gene (*si:dkey-67c22; ENSDARG00000055389*) and the *kcne1* paralogous gene (*AL807829*; *ENSDARG00000087204*) surrounding this additional *rcan1 (rcan1a)*. Vertebrate *Srrm1* and *Kcne1* are usually neighbours of *Rcan3* and *Rcan1* genes respectively. Moreover, the comparative genomic analysis links this region including the *rcan1a* gene in zebrafish with *RCAN3* and *RCAN1* regions in human genomic sequences. Therefore, this vertebrate organism bears four *rcan* genes: *rcan1b*, *rcan2*, *rcan3* and the additional *rcan1a*, which probably originated from a recombination of the *rcan1b* and *rcan3* genes.

Annotation of *Rcan2*, but not *Rcan1* and *Rcan3*, is absent in teleost fishes other than zebrafish, such as *Oryzias latipes* (medaka), *Tetraodon nigroviridis* (tetraodon), *Takifugu rubripens* (fugu), *Gadus morhua* (cod), *Gasterosteus aculeatus* (stickleback), *Xiphophorus maculatus* (platyfish) and *Oreochromis niloticus* (tilapia). Using the zebrafish *rcan2* gene sequence as a reference, we were not able to find homology with any genomic region for the teleost fishes analysed. However, all of them contain at least two copies of *Clic5*. Additionally, in all teleost fish, including zebrafish, while the *Runx1*-*Clic6*-*Rcan1* (ACD21) cluster has been maintained, *Rcan3* is located near *Nipal3*, but separated from *Clic4* and *Runx3*, fragmenting the ACD1 cluster. These characteristics suggest that some chromosomal rearrangements took place at different moments in the evolution of the teleost fish. These rearrangements affected the ACD1 cluster, which was fragmented, and the ACD6 cluster that lost *Rcan2* posterior to zebrafish divergence, while additional *CLIC* genes appeared.

*Anolis carolinensis* (anole lizard) also lacks *rcan2* and even its neighbour partner gene *enpp5*. Since all ACD clusters are present in other Sauria, such as *Pelodiscus sinensis* (Chinese softshell turtle), the *rcan2*-*enpp5* genomic region of this turtle was used to perform a comparative genomic analysis against the lizard genome sequence. We did not find any homologous region on the lizard genome, suggesting a posterior event to its divergence from the rest of Sauria, which gave rise to the loss of the *rcan2* and *ennp5* genes in this organism.

In the case of the primate Callithrix jacchus (marmoset), our search in the Ensembl database [32] retrieved six annotated RCAN genes: ENSCJAG00000002838, ENSCJAG00000012084, ENSCJAG00000020838, ENSCJAG00000010396, ENSCJAG00000034792 and ENSCJAG00000033745. Given their location in syntenic regions with human chromosome 21, 6 and 1, their relative position to RUNX genes and their homology with human paralogous genes, the ENSCJAG00000002838, ENSCJAG00000012084 and ENSCJAG00000020838 genes correspond to RCAN1, RCAN2 and RCAN3, respectively. Regarding the ENSCJAG00000010396 RCAN gene, it was named RCAN2 in a previous version of the Ensembl database (release 68, July 2012) [32]. However, it is located on chromosome 6, in a syntenic region to HSA 2, while the RCAN2 gene is located in HSA 6. For this reason, we consider the ENSCJAG00000010396 marmoset gene to be an additional RCAN gene or pseudogene highly similar to hRCAN2. Regarding the other two additional RCAN genes in marmoset (ENSCJAG00000034792 and ENSCJAG00000033745), they are located in non-assembled DNA scaffolds (GL287717.1 and GL288716.1, respectively). By means of a BLAST search using the megablast option [62], we were able to find a non-annotated region on marmoset chromosome 1 where these two scaffolds would be located. These two sequences could constitute two alternative transcript forms of the same novel RCAN gene. The origin of this novel additional RCAN (conserved as a gene or pseudogene) in this organism may be a recent gene duplication, probably of the RCAN1 gene, due to the closest similarity of its protein product to hRCAN1 protein (63–77% of amino acid identity). Therefore, our analysis indicates that there are 5 RCAN genes in the marmoset: RCAN1, RCAN2, RCAN3 and two additional RCAN genes, one of them similar to hRCAN1 and the other similar to hRCAN2, the origins of which may be recent duplication events.

Regarding *CLIC* genes, on the whole all mammals have the six *CLIC* genes. However, some *CLIC* genes are missing from some species. For instance, *Clic2* exists in the rat, but not in the mouse. Otherwise, for some species there are pseudogenes poorly annotated in the Ensembl database [32] as additional *CLIC* genes. For instance, *ENSCJAG00000000325*, a supposed marmoset additional *CLIC* gene, is actually a pseudogene homologous to the human pseudogene *LOC100420638* (gi:302486278, NG_026199.1) annotated at NCBI-Gene.

In summary, apart from the general trends analysed in the previous section, genomic evolution of the *RCAN* genes has been the result of several rearrangements and duplication events that have led to the gain and/or loss of *RCAN* gene copies in some organisms.

### Structure of Human *RCAN* Genes and Comparison with other Vertebrates

**Figure 3:**
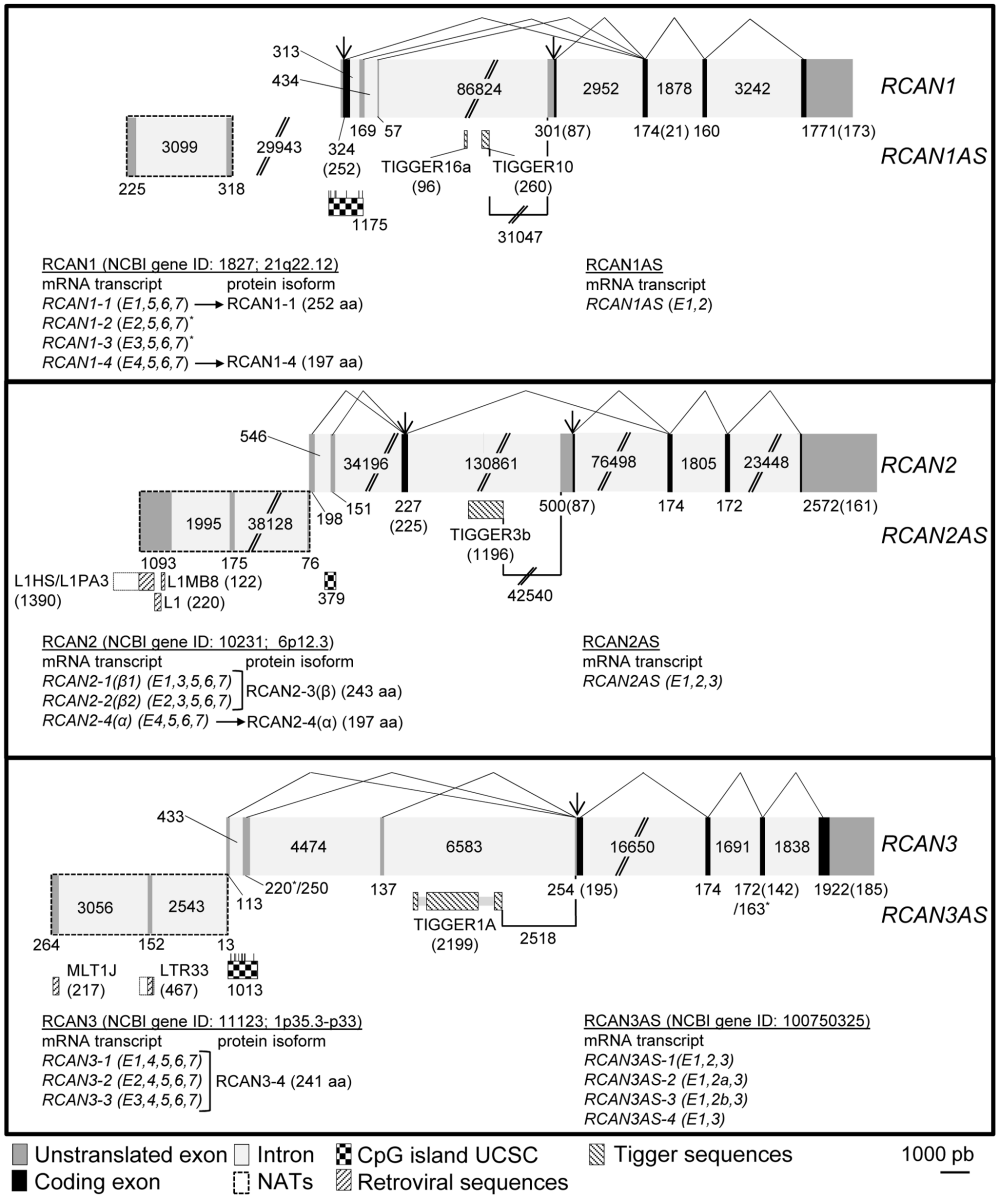
Gene structure comparison of human RCAN family members. The three members of the human *RCAN* gene family include seven exons, the last three being coding exons for which the encoded amino acid sequence is highly conserved between the three paralogous proteins (see Figure S3). Arrows indicate translation start sites, non-coding exons are represented in grey and coding exons in black, and exon order goes from left to right. Lines connecting exons indicate the different exon usage for each *RCAN* transcript form. Double lines in intron indicate omitted fragments (non-scaled intron length). Numbers indicate intron and exon sizes. Numbers in parenthesis indicate coding nucleotides within exons containing untranslated and translated regions. Vertical bars above CpG island associated to *RCAN1* and *RCAN3* correspond to the exactly positions of the methylation probes included in the methylation array. Asterisks in *RCAN1* mRNA forms (^*^) indicate that these transcripts have not been detected at protein level. Note that *RCAN3* exon numbering follows the new nomenclature outlined in Figure 4 and only the *RCAN3* mRNA transcripts that code for RCAN3-4 protein isoform are represented. Natural antisense transcripts (NATs) related to each *RCAN* gene (squared by dashed lines and where exons order goes from right to left) and regions with homology with transposable elements (in NATs and intron 3) are also shown.

Vertebrate RCAN proteins are highly conserved at their central and C-terminal regions, but they differ in their amino-terminal region (Figure S3). Moreover, when comparing the human *RCAN* gene structure, remarkably similar features (Figure 3) can be found apart from the presence of seven exons, the last three of them conserved in all the RCAN genes.

The human *RCAN1* is located on human chromosome 21 (HSA 21q22.12). This gene contains 7 exons, the first four being alternative and mutually exclusive first exons, whereas exons 5, 6 and 7 are common to all transcript forms (Figure 3, top panel). Four transcript forms have been identified for human *RCAN1* by 5′RACE [63], three of them are annotated in the RefSeq database [35] and all of them are included in the UCSC database [34]. Among these *RCAN1* transcripts, *RCAN1-1* (exons 1,5,6,7) and *RCAN1-4* (exons 4,5,6,7) are the predominantly expressed forms. These two transcripts encode for protein isoforms RCAN1-1 and RCAN1-4, respectively. RCAN1-1 isoform is constitutively expressed but subjected to up-regulation by glucocorticoids and vascular endothelial growth factor (VEGF) [64] and down-regulation by the Notch signalling pathway [65]. *RCAN1-4* transcription is induced by increases of intracellular calcium concentration, due to the presence of multiple NFATc and C/EBPβ binding sites in its promoter, and by estrogen hormones, among other stimuli [66], [67]. RCAN1-1 and RCAN1-4 isoforms are ubiquitously expressed, with abundant expression in adult heart, while RCAN1-1 is also expressed at high levels in fetal brain [63], [68]. By means of a comparative genomic analysis, we found that jawed vertebrates *RCAN1* shows high conservation of coding *RCAN1* regions and slightly less conservation of the 5′-untranslated region (UTR) and 3′-UTR (Figure S4A).

The *RCAN2* gene, which maps onto the human chromosome 6 (HSA 6p12.3) comprises 7 exons with exons 5, 6 and 7 being common to all transcript forms (Figure 3, middle panel). Similarly to the *RCAN1* gene, exons 1, 2 and 4 are mutually exclusive first exons (Figure 3). Three mRNA forms and two protein isoforms have been described in humans. Exon 3 is present in both RCAN2-1 (E1,3,5,6,7) and RCAN2-2 (E2,3,5,6,7) transcripts and although they contain a different 5′-UTR first exon they encode for a same protein product, RCAN2-3 (formerly RCAN2-β) protein. RCAN2-4 (formerly RCAN2-α) protein is encoded by *RCAN2-4* (exons 4,5,6,7). Transcripts *RCAN2-1* and *RCAN2-2* are ubiquitously expressed, with abundant mRNA levels in brain, heart, skeletal muscle and liver, while *RCAN2-4* has only been detected in brain [69]. *RCAN2-4* gene transcription is upregulated by thyroid hormone in human skin fibroblasts [69]. Our comparative genomic analysis of vertebrate *RCAN2* genes shows that *RCAN2* gene coding regions and the 5′ UTR of exon 4, which are only annotated for mammals and chicken genomes, are highly conserved in vertebrates (Figure S4B). Furthermore, it indicates that, while exons 1, 2 and 3 are conserved among mammals, they are only annotated in primates and the SLAGAN version of human-mouse alignment. The *RCAN2* 3′-UTR presents high sequence conservation among primates, horse and dog, while it is reduced in the case of rodents. For chicken, zebrafish and frog, this region seems not to be annotated (Figure S4B).

**Figure 4:**
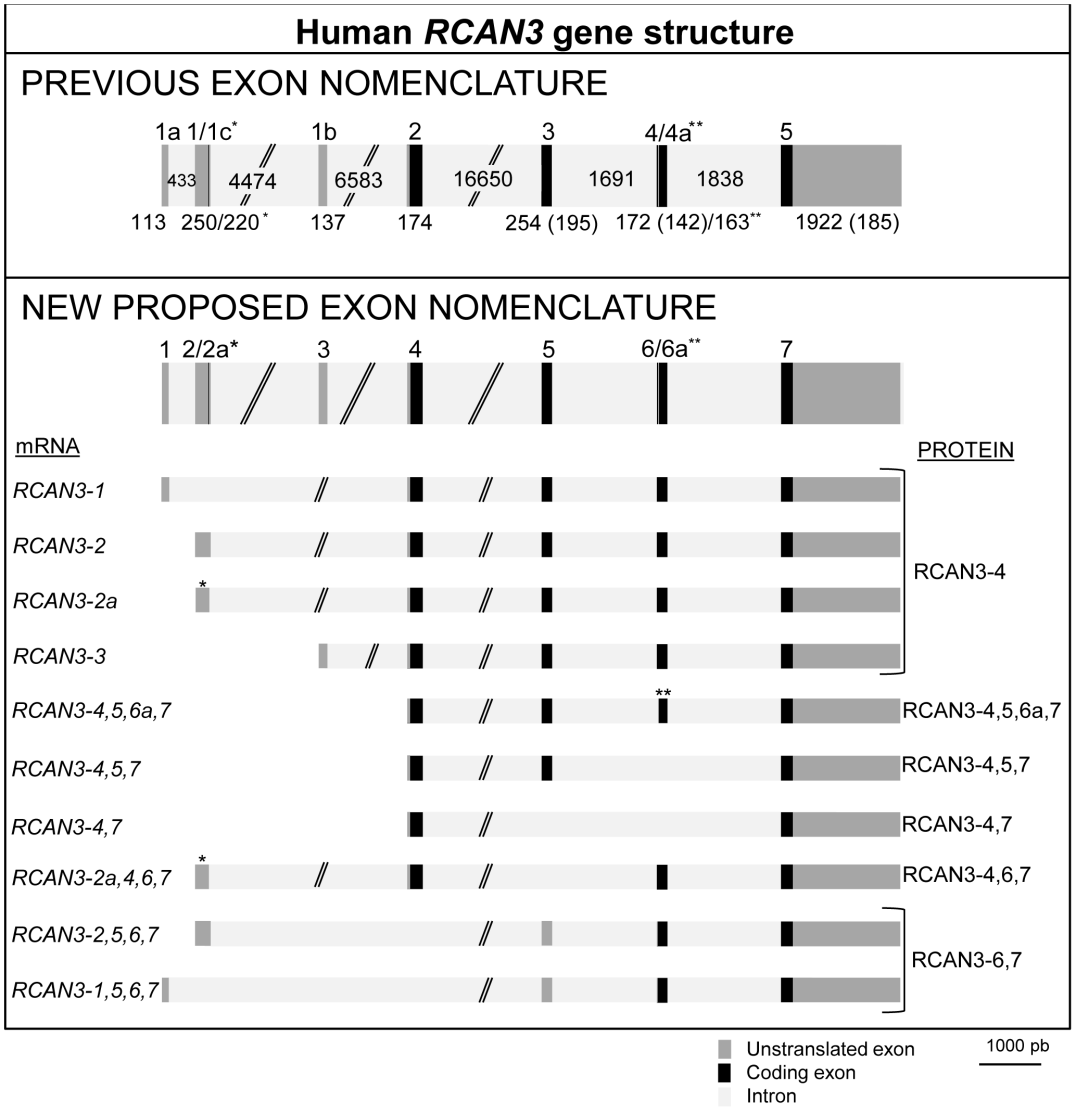
Human RCAN3 gene structure, alternative transcript forms, and protein isoforms. The scheme shows the new proposed exon nomenclature in comparison to that previously established and reviewed in Davies and colleagues [1] taking into account the recently described new exons [31] and those transcripts accepted in the RefSeq database [35]. Black rectangles correspond to coding exons, dark grey rectangles to non-coding exons (5′ and 3′ UTR) and light grey rectangles correspond to intron regions. All intron and exon sizes (in bp) are indicated and represented in scale, except for introns 2, 3 and 4. The size of the coding sequence in those exons that contain untranslated and translated regions is shown in parenthesis. Exon 2 transcription start site (TSS) (corresponding to exon 1 in the previous exon nomenclature) is the unique site that has been demonstrated by 5′ RACE. *RCAN3-1*, *RCAN3-2*, *RCAN3-2a* and *RCAN3-3* mRNA forms, all including coding exons 4, 5, 6 and 7, are translated into the same protein, named RCAN3-4, the longest known isoform for RCAN3 (241 amino acids) and the only one detected at the endogenous level. Asterisks indicate the presence of a particular exon in specific transcripts: *RCAN3-2a* and *RCAN3-2a,4,6,7* transcripts include the non-coding exon 2a, an in-frame shorter variant of exon 2, and *RCAN3-4,5,6a,7* transcript includes the coding exon 6a that lacks an in-frame 30 nt length segment of exon 6.

Concerning human *RCAN3*, located on chromosome 1 (HSA 1p35.3-p33), it also includes seven exons with the last four exons being mostly common to all known transcript forms. The first three exons are mutually exclusive and non-coding (Figure 3, bottom panel). Up to 21 alternative transcripts of human *RCAN3* have been described [27]–[31] but only ten of them have been accepted as completed mRNA in the RefSeq database [35]. To avoid the complex nomenclature of the different transcript forms of human *RCAN* genes we propose a novel classification of the *RCAN3* exons and of the different *RCAN3* accepted transcripts in the RefSeq database, according to the different exons being used [1] (Figure 4 and Table S2) (from now on exons and transcripts are referred to following the novel nomenclature proposed). Therefore exons 1a, 1, 1c, 1b, 2, 3, 4, 4a and 5 from the previous nomenclature correspond to the 1, 2, 2a, 3, 4, 5, 6, 6a, and 7, respectively, of the new nomenclature (Figure 4). Consequently, human *RCAN3* transcript forms *RCAN3-1, -2, -2a* and *-3* give rise to the same mature RCAN3-4 protein (RefSeq: NP_038469; UniProt: Q9UKA8-1; 241 amino acid) of about 35 KDa, the unique RCAN3 protein detected for human and mice so far [Unpublished data], [70]. The *RCAN3-4,5,6a,7* transcript contains a 10 amino acid deletion of the RCAN3-4 isoform due to an in frame 30 nt deletion at exon 6. Transcripts not containing exon 5 or/and 6 modify the open reading frame and therefore would code for proteins with no amino acid identity at the RCAN common central and C-terminal regions. It has been reported that the *hRCAN3* gene is constitutively and ubiquitously expressed, predominantly in heart, brain, small intestine, lung, testis, prostate and peripheral blood leukocytes (PBL) [27]–[31]. Our whole genome comparative analysis of the *RCAN3* gene (Figure S4C) revealed that the coding region of exon 4 is neither conserved nor annotated in *Xenopus laevis*. The *RCAN3* 5′-UTR exons (exons 1, 2/2a and 3) are annotated and conserved in primates, as has been previously reported [31], but they are not conserved in the other mammals. However, after performing an exhaustive search, we were able to find that exon 2 is also annotated in *Bos taurus* and *Mus musculus* (data not shown).

**Figure 5:**
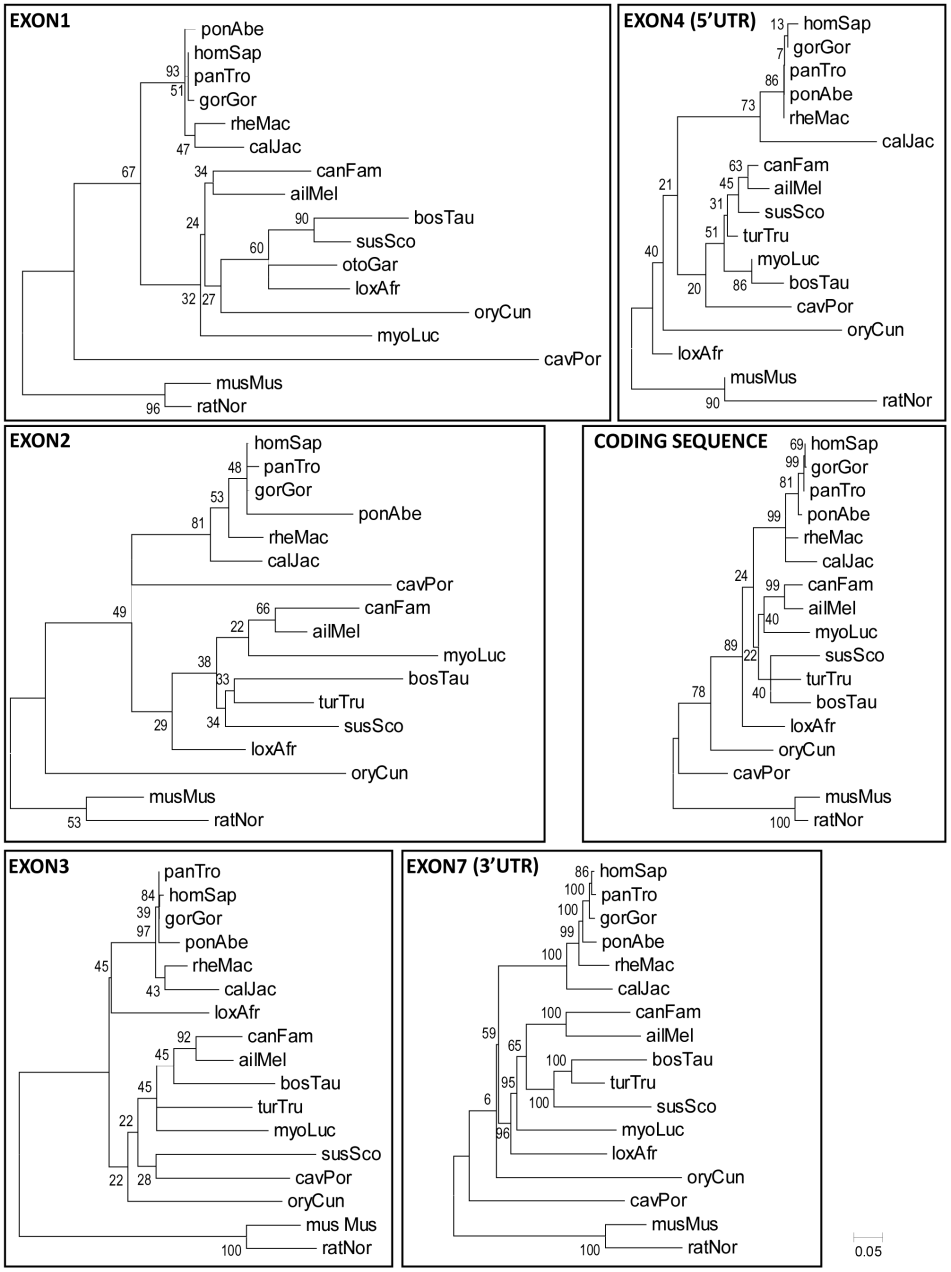
Phylogenetic analysis of the RCAN3 exons in vertebrates. The human *RCAN3* genomic sequences corresponding to first exons (exon 1, 2 and 3) and to exon 4 untranslated regions (5′ UTR), coding region (exon 4, 5, 6 and 7) and exon 7 untranslated region (3′ UTR) were compared with the sequences of several vertebrate *RCAN3* orthologs. DNA sequences for the different species were retrieved from the Ensembl database [32] and used for subsequent phylogenetic analysis, as described in the Material and Methods section. All primate genomic sequences appear as very closely related sequences, while rodent sequences (*Mus musculus*, *Rattus norvegicus* and *Cavia porcellus*) are the most divergent of all the mammals in all cases, except for exon 2, exon 3 and the short 5′UTR sequence on exon 4. The species studied and the genome sequence versions used were: *Homo sapiens* (v.GRCh37.p7 Feb 2009), *Pan troglodites* (panTro, Chimpanzee; v.2.1.4 Feb 2011), *Gorilla gorilla* (gorGor, v.3.1 Dec 2009), *Pongo abelii* (ponAbe, Orangutan; v.2 Sep 2007), *Macaca mulatta* (rheMac; v.1.0 Feb 2006), *Callithrix jacchus* (calJac, Marmoset; v.3.2.1 Jan 2010), *Cavia porcellus* (cavPor, Guinea Pig; v.3 Mar 2008), *Sus scorfa* (susSco, Pig; v. 10.2_Aug 2011), *Mus musculus* (musMus, Mouse; v.37 Apr 2007), *Rattus norvegicus* (rarNor, Rat; v.3.4 Dec 2004), *Oryctolagus cuniculus* (oryCun, Rabbit; v.2 Nov 2009), *Ailuropoda melanoleuca* (ailMel, Panda; v.1 Jul 2009), *Canis lupus familiaris* (canFam, Dog; v.2.0 May 2006), *Bos taurus* (bosTau, Cow; v.3.1 Nov 2009), *Tursiops truncates* (turTru, Dolphin; v.1 Jul 2008), *Loxodonta africana* (loxAfr, Elephant; v.3.0 Jul 2009), *Myotis lucifugus* (myoLuc, Microbat; v.2.0 Sep 2010). Numbers at the tree nodes correspond to bootstrap values. The scale bar in the bottom refers to the branch lengths and the number indicates substitutions per site.

In order to deepen our understanding of the genome evolution of the *RCAN3* 5′ UTR (exons 1, 2, 3 and part of 4) and 3′ UTR (part of exon 7) of the *RCAN3* gene, we performed a phylogenetic study of these regions in several mammals (Figure 5). For this analysis, annotated human sequences were used to delimitate 5′ UTR, 3′ UTR and coding exons in other species, except for exon 2, where the mouse annotated sequence was used, since it spans further upstream than the human sequence (Figure 5). The results obtained indicate that the UTR of *RCAN3* exons in rodents (ratNor, musMus and cavPor) are more divergent than in the rest of the mammals analysed, except for exon 3 and exon 4 5′UTR in *Cavia porcellus* (cavPor). Furthermore, these UTRs are highly conserved between primates, as they appear strongly clustered in the phylogenetic tree. Moreover, non-coding exons (exons 1, 2/2a and 3), 5′ UTR of exon 4 and 3′ UTR of exon 7 show higher sequence divergence than coding region as the length of the branches indicates (Figure 5), but all of them behave in a similar manner. Therefore, species *RCAN3* comparison suggests a parallel evolution between coding and non-coding exons.

In summary, after analysing the *RCAN* gene structure, and renaming *RCAN3* exons, we can conclude that all the genes of this family have 7 exons, exons 5 to 7 being common to all transcripts and sharing high amino acid identity in their encoded proteins (Figure S3). In addition, the first three/four exons of each *RCAN* gene are mainly mutually exclusive first exons of transcript forms, which are regulated by different proximal promoter regions.

### Natural Antisense Transcripts and Transposon Sequences Associated with *RCAN* Genes

Recently, four natural antisense transcripts (NATs) that partially overlap the *RCAN3* gene have been described [31]. These transcripts are encoded by the same gene, which is named *RCAN3AS* and includes three exons and two introns (Figure 3, bottom panel). Alternative splicing of the second exon generates the transcripts. These transcripts have not been shown to be translated into proteins. In this context, we decided to search for similar NATs in both *RCAN1* and *RCAN2* genes. One gene (*BC042616*, corresponding to *RP11-795J1.1* (*ENSG00000236466*) in the Ensembl database) overlapping exon 1 on *RCAN2* and one EST (*DA403464*) located at the 5′ region of the transcription start site (TSS) of the *RCAN1* exon 1, both with similar characteristics to *RCAN3AS* transcripts, have been identified. Both NATs are located upstream of the corresponding *RCAN* genes and transcribed in the opposite sense to the *RCAN* corresponding gene (Figure 3, where NATs are shown squared by dashed lines and exons are numbered from right to left). Therefore, we suggest naming these genes following the same nomenclature proposed by Facchin and colleagues for the NATs associated to *RCAN3*, *RCAN3AS* [31]. Thus, *RCAN1AS* and *RCAN2AS* designate the *RCAN1* and *RCAN2* antisense genes.

As Figure 3 shows in more detail, it is not difficult to draw parallels between *RCAN3AS* and *RCAN2AS* transcripts; both include two introns, three exons and the first exon partially overlapping with the exon 1 of the corresponding *RCAN* gene (10 nt in *RCAN3* and 16 nt in *RCAN2*).

Regarding *RCAN1AS,* the scenario slightly differs from the previously described *RCAN*-associated NATs (Figure 3). One antisense EST for the *RCAN1* gene (GenBank ID: *DA403464*, from human thalamus) has been described that it is not included in the RefSeq database [35] as a gene record. The transcript corresponding to this EST seems to be encoded by a gene that includes at least two exons and one intron, however we can hypothesize that an additional exon can be found overlapping exon 1 of the *RCAN1* gene, but it has not been identified yet. It is noteworthy that this NAT has been described as capable of regulating *RCAN1* gene expression and patented as a putative agent for the treatment of Down’s syndrome (patent WO/2010/151674 A2). Due to this *RCAN1* NAT structure and location resemblance to the *RCAN2* and *RCAN3* NATs, together with its described functional effect, we propose the *DA403464* EST as an *RCAN1AS* transcript (Figure 3).

As Figures S4A and S4B show, *RCAN1AS* and *RCAN2AS* are not annotated as transcribed mRNA for any of the organisms analysed other than human, whereas *RCAN3AS* is annotated in primate alignments with human sequences and only the *RCAN3AS-E3* is annotated between human and mouse in the SLAGAN alignment. Manual alignment of the corresponding genomic sequences of several organisms with respect to the human *RCANAS* transcripts was performed (Figure S5). The results indicate that all species which do not contain sequencing gaps in these regions share more than 50% of nucleotide sequence identity with human *RCANAS* transcripts, these being almost identical in primates. In addition, by means of BLAST search using the BLASTn option [36] with these three *RCANAS* against the EST database, we found different ESTs from several organisms with high sequence identity to the human *RCAN2AS-E3,* but poor sequence identity with *RCAN1AS* and *RCAN3AS* transcripts and only 10–15% of query coverage, indicating that at least *RCAN2AS* is transcribed in several organisms.

In order to go further into the origin and the nature of the antisense transcripts, we compared the *RCAN* NATs with the sequences available in the Repbase database of repetitive DNA elements by using the CENSOR web server tool [40], [71]. Our results indicate that the *RCAN3AS* nucleotide sequence presents more than 85% sequence identity with the LTR-retrotransposons MLT1J and LTR33, and that *RCAN2AS* presents a similar identity with LINE-1 retrotransposons (Figure 3) and, remarkably, also with the *LOC340211* gene (similar to LINE-1 reverse transcriptase homolog at human chromosome 22; NM_001012976). This suggests a possible retrotransposon origin for these transcripts. Nevertheless, for the *RCAN1AS* nucleotide sequence analysis we did not retrieve any homology with retrotransposon sequences. When analysing the presence of repetitive elements within the *RCAN* genes by using the Repbase Database [71] and the CENSOR web server tool [40], we also detected the presence of DNA sequences from the Tigger DNA-transposon in intron 3 of the three *RCAN* genes (Figure 3). In particular, Tigger1A sequence located in the *RCAN3* intron 3 is found intact and conserves its open reading frame (ORF), which codes for a transposase, and the two inverted repeats (IRs) [72], while in *RCAN2* only the central ORF is conserved. In *RCAN1* the Tigger sequence is highly degenerated and only a few fragments are conserved. Interestingly, Tigger sequences in *RCAN1* and *RCAN2* are highly conserved in primates, but are not present in other mammals (data not shown).

Therefore, all human *RCAN* genes seem to have associated NATs, transcribed in the opposite sense, which partially overlap with the corresponding *RCAN* gene in case of *RCAN2* and *RCAN3*. These results suggest that the *RCAN* promoters could function as bidirectional promoters regulating gene expression of both *RCAN* and *RCANAS* genes. Genomic sequences that code for these NATs present a high homology among mammals. Moreover, analysis for repetitive sequences in *RCAN* genes revealed a possible transposon nature of these NATs and the Tigger sequences in the intron 3 of the three human *RCAN* genes.

### Analysis of *RCAN* Gene Promoters

As mentioned before, *RCAN1* and *RCAN2* gene expression regulation has been studied to some extent, but the functional regulation of the *RCAN3* gene is still unknown. *In silico* analysis predicted that the *RCAN3* transcripts that bear exon 1, 2 or 2a as the first exon are driven by TATA-less promoters, while transcripts starting with exon 3 are regulated by a promoter containing a TATA box [27], [31]. Some TATA-less *RCAN* transcripts have their transcription start site (TSS) within a CpG island, such as the TSS of exon 2 of the three *RCAN* genes and the TSS of exon 1 of *RCAN1* (Figure 3). Additionally, these CpG islands are surrounded by regions with high CG content, so these *RCAN* transcripts are likely to be regulated by a promoter susceptible to methylation.

To examine this, we explored the methylation status of the *RCAN*-associated CpG islands by using the data on the “Infinium HumanMethylation450 BeadChip (Illumina)” methylation array (data not shown). All the probes mapping to CpG islands associated with *RCAN1* and *RCAN3* (Figure 3, vertical bars above CpG islands) presented an unmethylated status in several normal and cancer human cell lines (data not shown), suggesting that these regions may be transcriptionally active. For the *RCAN2* promoter CpG island, unfortunately none of the probes included on the array mapped to the CpG island associated to *RCAN2*, so its methylation state and expression activity remain unknown.

**Figure 6:**
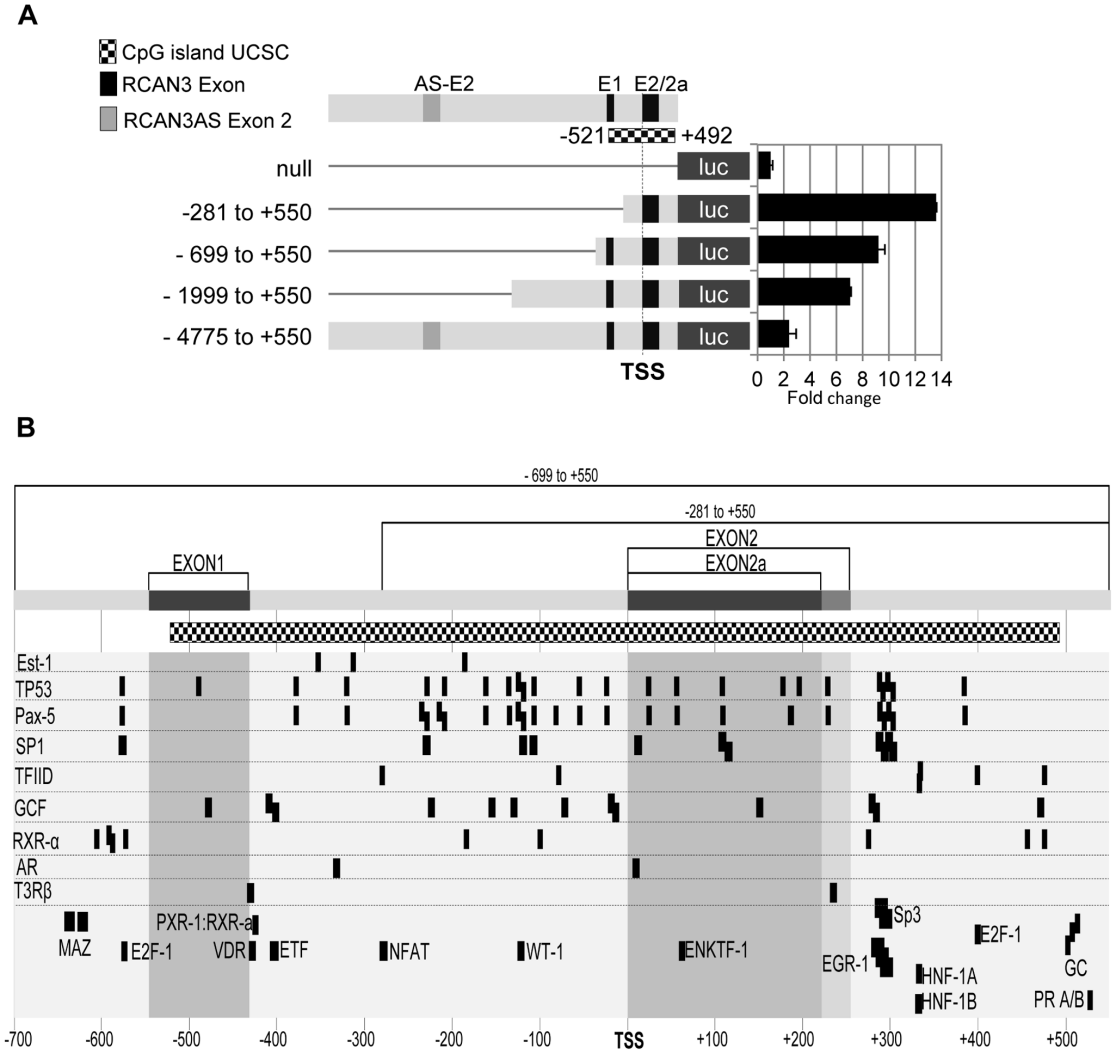
Transcriptional activity and in silico prediction of TFBS along the 5′ region of the RCAN3. (A) Upper panel, schematic representation of serial DNA regions 5′ flanking the transcription start site (TSS) of the *RCAN3-2/2a* transcripts cloned into a pGL3-luc promoterless reporter vector (see details in Materials and Methods). The 5′ flanking region of each construct is shown in bp referred to the TSS of exon 2. TSS (+1) is indicated with a vertical dashed line. Black boxes upstream of the luciferase gene correspond to *RCAN3* non-coding exons 1 (E1) and 2/2a (E2/2a), and the dark grey box corresponds to exon 2 of RCAN3AS (E2AS). Squared dotted box indicates the CpG island location surrounding 2/2a exon. Lower panel, luciferase reporter assays in HEK 293T cells transfected with 30 fmol of each construct. Luciferase/Renilla activity values are presented as a fold-change relative to the activity of the empty vector. The graph shows the mean ± standard deviation of three independent experiments performed in triplicate. (B) *In silico* prediction of human TFBS in −700 to +550 region respect to the TSS of exon 2 using PROMO software [41], [42]. Exons 1 and 2/2a relative position are indicated above as dark grey boxes and the CpG island as a squared dotted box. Guide number below indicates distances with respect to the TSS of *RCAN3-2/2a* transcripts. Predicted TFBS are indicated as dark boxes with the name of the transcription factor next to the boxes or left or right at the same level when multiple predictions for the same factor.

Assuming that this unmethylated conformation of the *RCAN3*-associated CpG correlates with a transcriptional activation of the gene, we analysed this hypothesis by using luciferase reporter gene assays. Different DNA regions 5′ flanking the TSS of exon 2 of *RCAN3* and including the CpG island (Figure 3 and 6A) were subcloned and analysed to assess their transcriptional activity (Figure 6A). All the constructs promoted luciferase gene expression at different levels. The construct harbouring the fragment of CpG island that contains exon 2 but not exon 1 (−281 to +550 nt, where +1 is the TSS of exon 2) gave the highest transcriptional activity. The addition of *RCAN3* exon 1 or more upstream sequences reduced luciferase transcriptional activity. These results suggest the presence of transcription factor binding sites (TFBS) or DNA conformational changes on *RCAN3* exon 1 and upstream sequences that may act as transcriptional repressors of *RCAN3* transcripts starting at exon 2 or 2a.

To predict the presence of putative TFBS in this human *RCAN3* proximal promoter, we performed *in silico* analysis using the PROMO software with *Homo sapiens* weight matrices [41], [42]. The analysis of the *RCAN3* CpG island (−521 to +492 nt; Figure 6A, see dotted bar) predicted many TFBS for SP1, TP53, and PAX5 in the −281 to +550 *RCAN3* respect to the TSS at exon 2/2a (Figure 6B). TFBS for some additional transcription factors (NFATc, MAZ, E2F-1, VDR and ETF) that were not present in the region −281 to +550 were predicted in sequences further upstream (−699 to −282).

We also performed a multispecies prediction of TFBS on the *RCAN3*-associated CpG island in the DNA sequence of 18 eutherian mammals using eutherian weight matrices for the prediction. Figure S6 shows some TFBS such as TP53 and PAX5 binding sites highly conserved among all the organisms analysed. This TFBS conservation through evolution reinforces the likelihood of a functional role of the CpG island region in the modulation of *RCAN3* gene expression.

Our results suggest that the genomic structure and regulation of the *RCAN* gene family has been conserved during mammalian evolution. Furthermore, we have shown that the *RCAN3*-associated CpG island, where exon 1 and 2/2a are located, is transcriptionally active and may be involved in regulating *RCAN3* gene expression in different cellular conditions. Further studies should be performed to understand how *RCAN3* gene expression is regulated.

## Discussion

Here we look in depth at the evolution of the *RCAN* gene family in jawed vertebrates, their human gene structure and regulatory elements involved in human *RCAN* gene expression in order to improve our knowledge of this gene family.

Regarding *RCAN* evolution in jawed vertebrates, *RCAN* genes, together with *RUNX* and *CLIC* genes, form part of what have been named ACD clusters [25]. Taking this into account we decided to look more carefully at *RCAN*, *RUNX* and *CLIC* evolution to extend our knowledge about *RCAN* genes.

In chordata invertebrates, Urochordata (*Ciona intestinalis*; sea squirt) and Cephalochordata (*Branchiostoma floridae*; amphioxus), the search for human *RUNX, CLIC* and *RCAN* orthologs indicated the presence of a unique copy for these genes, that are located separately.

Regarding *RUNX* genes in vertebrates, the two living representative organisms of jawless vertebrates, Atlantic hagfish (*Myxine glutinosa*) and sea lamprey (*Petromyzon marinus*), have two orthologs of human *RUNX,* named *RunxA* and *RunxB* [53], [54], while jawed vertebrates carry three copies. It had been previously reported that the ancestral chordate runt domain, the precursor of human *RUNX* genes, underwent a primary duplication, generating *Runt* and the ancestral *Runx* gene (Figure 1, *ancRunx* and *ancRunx’*), followed by a posterior triplication of *Runx* that was the origin of *Runx1, Runx2* and *Runx3*, present in all jawed vertebrates [73]. Given this fact, *Runt* (*ancRunx*) probably lost functionality during jawed vertebrate evolution and disappeared. This hypothesis is concordant with ours, which goes one step further and fixes the first duplication event as the 1R-WGD and the triplication step as the 2R-WGD followed by one segmental duplication event, as the origin of the actual three *RUNX* genes present in jawed vertebrates (Figure 1).

As regards the *CLIC* genes, the data obtained from the phylogenetic analysis (Figure S2 and ENSGT00550000074477 from Ensembl [32]) indicates a divergence at the very early stages in vertebrate evolution between *LpClic1*/*CLIC1*/*CLIC3* and *LpClic5*/*CLIC4*/*CLIC5*/*CLIC6* ancestors and, as the lamprey is considered to be a “living fossil” [74], we consider *Lp*C*lic1* and *LpClic5* genes to be the most representative of these ancestral genes, regardless of whether lamprey has undergone one or two rounds of WGD.

Concerning *RCAN* genes, our attempt at finding *RCANs* in several databases using human *RCAN* or *Caenorhabditis elegans Rcn-1* sequences as templates, did not initially report any homologous protein or gene in Atlantic hagfish and sea lamprey. One possibility is that both copies of the ancestral *Rcan* probably originated after the 1R-WGD were lost in jawless vertebrates. Nevertheless, we cannot discard their existence for several reasons. In the case of hagfish, its genome has not been sequenced yet. In the case of sea lamprey (*Petromyzon marinus*), despite its genome being considered to be complete [24], it still contains gaps (GenBank Assembly ID: GCA_000148955). Moreover, the sequencing has been performed from the genomic DNA derived from the liver of a single adult specimen. It has been reported that agnathans undergo extensive genomic rearrangements in the early embryonic development [75]. Thus, it is possible that they bear *RCAN* genes in the genome, but they disappear in adult specimens and/or in specific adult organs as a result of these extensive rearrangements. In fact, we were able to find a possible ortholog of human *RCAN* in Arctic lamprey (*Lethenteron camtschaticum*), another lamprey species. Regarding jawed vertebrates, apart from the three *RCAN* genes already described, we also identified two additional *RCAN* genes for the marmoset primate and the loss of one *RCAN* copy in teleost fish except zebrafish.

Considering all this available data, we propose a new hypothesis for a plausible explanation of the evolution of *CLIC*, *RUNX*, and *RCAN* genes, different to the previous proposal in which evolution of these genes in ACD clusters originated from successive segmental duplications and rearrangements during the two rounds of whole genome duplication [25]. This novel hypothesis is graphically described in Figure 1 and S1. Briefly, invertebrates only harbour unique copies of *Clic*, *Runx*, and *Rcan,* which are independently located, suggesting that they are not functionally related (Figure 1, invertebrates). The first round of genome duplication (1R-WGD) produced an additional copy for the *Clic*, *Runx*, and *Rcan* genes. Jawed vertebrates (gnathostomes) lost one of the two copies of the *Runx* and *Rcan* genes generated after the 1R-WGD. Afterwards, one copy of the *Clic* gene (*ancClic’*, the ancestor of the current human *CLIC4*, *CLIC5*, and *CLIC6*) was clustered together with the ancestral copies of *Runx* and *Rcan* genes (*ancRunx’* and *ancRcan’*, respectively). Posterior to ACD clustering, an additional round of whole genome duplication (2R-WGD) generated the ACD21 cluster, and a subsequent segmental duplication event between chromosome 1 and 6, probably originated the definitive ACD1 and ACD6 clusters. This idea is reinforced by the presence of up to 35 homologous genes located around ACD1 and ACD6 clusters (Figure 2 and Table S1).

The maintenance of the clustered distribution of these genes among jawed vertebrates evolution suggests a possible cooperation of ACD clustered genes. In fact we have observed that this functional cooperation is plausible. For instance, a possible cooperation among ACD clustered genes could be inferred from their role in immune responses and skeletogenesis. As far as the immune response is concerned, the role of CLIC [76], [77] and RCAN [78], [79] proteins in innate immunity has been extensively studied in several organisms. Both proteins are involved in Toll-like receptors (TLR) signalling and inflammation. Moreover, the role of RUNX proteins in innate immune responses is evident from their involvement in macrophage differentiation, monocyte migration and dendritic cells (DC) maturation [80]. Likewise, RUNX [81] and RCAN [82], [83] proteins have been described as participating in adaptive immunity. On the other hand, the most important evidence for cooperation between these three families of proteins is their participation in osteoblast differentiation and/or function and, subsequently, in bone formation [84]–[86]. Thus, the three genes in ACD clusters seem to play essential roles in several processes in vertebrates and this suggests that their clustered organization and further maintenance throughout evolution is due to functional requirements.

Concerning human *RCAN* gene structure, the recent discovery of additional human *RCAN3* exons [31] instead of the 5 exons previously described, has shown the existence of seven exons in all *RCAN* genes. Therefore we decided that it would be interesting to rename *RCAN3* exons to improve and facilitate the *RCAN* gene structure, transcript forms and isoforms analysis (Figure 4 and Table S2). With this novel exon nomenclature, exons 5 to 7 of *RCAN* show a high amino acid identity and they are conserved in vertebrates [16]. On the topic of UTR regions, all human *RCAN* genes harbour several non-coding exons that are mutually exclusive first exons in different *RCAN* transcripts. In *hRCAN3* at least three 5′ non-coding exons (E1, 2/2a and 3) that are mutually exclusive have been described (Figure 4). In an attempt to explore the evolution of the *RCAN3* UTRs, we performed a phylogenetic study using the corresponding genomic sequences of these exons in other organisms. Our analysis indicates that the UTR sequences are very close in primates, while in rodents the sequences seem to be the most divergent (Figure 5). In addition, if we compare non-coding *versus* coding exons, their phylogenetic trees are similar. Therefore, we can hypothesize that non-coding exons may be present in the primordial jawed vertebrate form of *Rcan3*, which originated after a segmental duplication event (Figure 1, see Segmental Duplication), suggesting that they can be found in all mammals and probably in other non-mammalian vertebrates. This idea can similarly be inferred from the genomic comparison of 5′ UTR exons of *Rcan1* and *Rcan2* presented in Figure S4.

Our gene structure analysis of human *RCAN* genes also unravels several features that could be related to its gene expression regulation. All the genes include a CpG island in at least one of the first exons of each gene. Regarding the *RCAN3* and *RCAN1* genes, exon 1 and 2 (and 2a in *RCAN3*) are included in a CpG island, but these exons are mutually exclusive in transcript forms. Our results show that these CpG island sequences associated with *RCAN1* and *RCAN3* genes are in an unmethylated state which probably points to a potential role in transcriptional activation. In addition, we also show that the CpG island associated to *RCAN3* exon 1 and 2/2a is transcriptionally active (Figure 6A). The addition of surrounding 5′ flanking regions to this CpG island negatively regulated its transcription. An *in silico* analysis of TFBS present in the proximal promoter region of exon 2/2a identified many SP1, PAX-5 and TP53 putative TFBS (Figure 6B and S6), some of them usually abundant in CpG islands [87]. Further experimental approaches should be used to determine the relevance and functional role of these TFBS in order to untangle the transcriptional regulation of *RCAN3*.

A detailed genomic sequence comparison of human *RCAN* genes allowed us to identify the presence of antisense transcripts upstream of *RCAN1* and *RCAN2* genes that have similar characteristics to those that had previously been described for *RCAN3* (Figure 3, squared in dashed lines, and Figure S4). Interestingly, in those cases that the genomic region corresponding to these *RCAN* antisense transcripts is annotated, there is a high sequence identity among mammals (Figure S5) which indicates that they could influence or modulate *RCAN* gene expression. This influence has already been demonstrated for *hRCAN1AS*, which regulates *hRCAN1* gene transcription in HepG2 and Vera cell lines (patent WO/2010/151674 A2). The functional role of this putative *RCAN1* NAT suggests the possible existence of a complex fine-tuning regulation of NAT and *RCAN* genes. Furthermore, the overlapping nucleotide sequences observed between the first exon of *RCAN* genes, at least for *RCAN2* and *RCAN3*, and NATs suggest the possibility that the gene expression regulation of both NAT and *RCAN* genes is carried out by a bidirectional promoter [88]. Interestingly, this bidirectional promoter is in close proximity or overlapping with the CpG island of the *RCAN* gene, a feature characteristic of bidirectional promoters [89]. It is also worth noting the presence of retrotransposon sequences, at least in *RCAN2AS* and *RCAN3AS*, which suggest an antique retrotransposition in an ancient predecessor of the three NATs of the *RCAN* genes (before the 2R-WGD), which was maintained in posterior duplications with some degree of divergence. DNA-transposition in the *RCAN* gene ancestors could also have contributed to the origin of the Tigger sequences found in intron 3 of the three *RCANs* (Figure 3). Tigger sequences have been maintained nearly intact in primates for *RCAN2* and *RCAN3.* These transposon sequences could be a reminiscence of an ancient event without a current function, or instead they could contribute to modulating transcription of precise mRNA forms in primates by conferring additional TFBS or a specific DNA structure.

In summary, our analysis contributes to improving the knowledge of *RCAN* gene evolution by providing evidences for a segmental duplication that would have been the origin of the current *RCAN2* and *RCAN3* genes in jawed vertebrates and on ACD clustering evolution and cooperative function. In addition, we have analysed *RCAN* gene structure and its NATs neighbours, and looked at the molecular mechanisms involved in RCAN gene expression regulation.

This improved knowledge of *RCAN* genome evolution and of the structural and functional elements present in the *RCAN* genes that could be involved in gene expression regulation, post-transcriptional modification and translation, provides novel clues to understanding the functional relevance of RCAN proteins in different physiological scenarios [2]–[6], [82], [83].

## Supporting Information

Figure S1 **Alternative proposed scenarios for the evolution of Runx, Clic and Rcan genes in sea lamprey (Petromyzon marinus).** Evolutionary hypothesis for *Runx*, *Clic*, *Rcan* genes in sea lamprey, considering that sea lamprey only underwent the first round of WGD (A) or that it also underwent the second round of WGD (B). Estimated times of 1R-WGD and 2R-WGD were obtained from *Vienne et al.* [58]. Thicker arrows indicate gene duplication events and the black-framed boxes correspond to gene losses. Abbreviations: anc, ancestral; agn, agnathans; gna, gnathostomes; Mya, Million Years Ago; WGD, Whole Genome Duplication.
(PDF)

Figure S2 **Phylogenetic analysis of CLIC transcripts.** Coding DNA sequences (CDS) of the known functional Lamprey *Clic* (named *LpClic1* and *LpClic5*) were retrieved from the Ensembl database [32] and used for subsequent phylogenetic analysis, as described in the Material and Methods section, together with all human, mouse, rat, sea squirt (*Ciona intestinalis*) and amphioxus (*Branchiostoma floridae*) *CLIC* CDS (*hCLIC*, *mClic*, *rClic, CiClic and BfClic*; respectively). Note that the Ensembl database contains three lamprey *Clic* genes: *LpClic1*, *LpClic5* and *LpClic6*. Since the *LpClic6* sequence is incomplete, it has been excluded from the analysis. The evolutionary tree obtained shown here relates *LpClic1* with human and rodent *CLIC1/3* and *LpClic5* with human and rodent *CLIC4/5/6*. Ensembl names for each transcript and Uniprot reference for *Branchiostoma floridae Clic* are indicated). Numbers at the tree nodes correspond to bootstrap values. The scale bar in the bottom refers to the branch lengths and the number indicates substitutions per site.
(PDF)

Figure S3 **Alignment of human RCAN proteins.** (A) Schematic representation of RCAN proteins structure, indicating the coding exons. The last three exons (exon 5, 6 and 7, following the novel nomenclature proposed here for RCAN3) are common to all RCAN isoforms; and the variable exons result mainly from alternative transcription start site (TSS) usage. GSK3β (*) and MAPK, BMK1 or DYRK1A (†) phosphorylation sites within the FLISPP motif, important in RCAN regulation, are indicated. Additionally to these phosphorylation sites described to be common to all human RCANs, it has been recently characterized an additional site in RCAN3 (Ser 203) and RCAN1 (Ser 218) that are able to be phosphorylated *in vivo* by CK2α [15]. (B) Table indicates percentage of amino acid conservation between regions common to all human RCAN proteins (encoded by exon 5 to 7, according to the new proposed nomenclature). (C) Protein sequence alignment among the different protein isoforms encoded by human *RCAN* genes protein RefSeq acc. number: RCAN1-1, NP_004405.3 (252 aa); RCAN1-4, NP_981963.1 (197 aa); RCAN2-3, NP_005813.2 (197 aa); RCAN2-4, NP_001238902.1/NP_001238903.1 (243 aa); RCAN3-4, NP_038469.1/NP_001238906.1/NP_001238907.1/NP_001238908.1 (241 aa). All of them share exons 5 to 7, according to the new proposed nomenclature. Some conserved residues appear even in the protein region encoded by the first exon, which may be important for its functional activity and/or regulation. Grey intensity shade increases with sequence conservation (50, 80 or 100% of amino acid conservation). Numbers correspond to amino acid positions for each protein.
(PDF)

Figure S4 **Comparative genomic sequence analysis of RCAN genes.** Alignment plots of *RCAN1* (A), *RCAN2* (B) and *RCAN3* (C) orthologs against human *RCAN* genes created by the VISTA tool of the UCSC browser [34], [52] using the Feb.2009-GRCh37/hg19 genome version for primates and mouse (SLAGAN alignment) and Mar.2006-NCBI36/hg18 for the other organisms. The vertebrate organisms used in the alignments against human sequences were: *Pan troglodites* (panTro), *Pongo abelii* (ponAbe), *Gorilla gorilla* (gorGor), *Callithrix jacchus* (calJac), *Macaca mulatta* (rheMac), *Mus musculus* (musMus), *Equus caballus* (equCab), *Canis lupus familiaris* (canFam), *Rattus norvegicus* (ratNor), *Gallus gallus* (galGal), *Danio rerio* (danRer), and *Xenopus tropicalis* (xenTro). The genome versions are indicated in the Material and Methods section. The chromosomal scale and base pair position guide are represented below the global comparative alignment in each assembly. All alignments are PROLAGAN alignments except when specified otherwise. In plots, darker blue indicates coding regions; lighter blue, untranslated regions (UTR) and pink, non-coding DNA sequence. 5′ UTR and 3′ UTR regions are conserved in primates and, to a lesser extent, in other mammals, although they are not always annotated. *RCAN* RefSeq and UCSC transcripts are shown below the alignment. Arrows in transcripts indicate the sense of gene transcription and, therefore, the order of the exons (darker blue boxes). UCSC registered CpG islands are indicated below in green. (A) Comparative genomic analysis of vertebrate *RCAN1* genes relative to the human gene (NCBI Gene ID: 1827). The natural antisense transcript (NAT) *DA403464* is registered as human UCSC EST. (B) Comparative genomic analysis of vertebrate *RCAN2* genes relative to the human gene (NCBI Gene ID: 10231). *BC042616* gene (*RCAN2AS*) is registered as UCSC gene. (C) Comparative analysis of the vertebrate *RCAN3* genes relative to the human gene (NCBI Gene ID: 11123). *RCAN3* transcripts and *RCAN3AS* NATs, accepted as RefSeq genes, are shown.
(PDF)

Figure S5 **Multi-species alignment of human RCAN1, RCAN2 and RCAN3 natural antisense transcripts: RCAN1AS, RCAN2AS and RCAN3AS.** Pip-type graph conservation profile of human *RCAN1AS* (*DA403464* EST), *RCAN2AS* (*BC042616* gene) and *RCAN3AS* (NCBI Gene ID: 100750325; [31]) in several eutherian mammals. Sequences were obtained using the genomic comparison tool of the Ensembl database [32] and alignments were generated and visualized by zPicture software [39]. Asterisk (*) indicates the region of exon 1 of *RCAN3-1* transcript that was manually included to the alignment due to the impossibility of aligning sequences shorter than 19 nt (*RCAN3AS* exon 1 length is only 13 nt). Non-aligned regions correspond to gaps in the genome sequence. Species and genomes assemblies used for the analysis were: *Homo sapiens* (v.GRCh37.p7 Feb 2009), *Pan troglodites* (panTro, Chimpanzee; v.2.1.4 Feb 2011), *Gorilla gorilla* (gorGor, v.3.1 Dec 2009), *Pongo abelii* (ponAbe, Orangutan; v.2 Sep 2007), *Nomascus leucogenys* (nomLeu1.0, Gibbon; v. Jan 2010), *Macaca mulatta* (rheMac; v.1.0 Feb 2006), *Callithrix jacchus* (calJac, Marmoset; v.3.2.1 Jan 2010), *Tarsius syrichta* (tarSyr, Tarsier; v.1 Jul 2008), *Microcebus murinus* (micMur, Gray Mouse Lemur; v.1 Jun 2007), *Otolemur garnettii* (otoGar, Bushbaby; v.3 Mar 2011), *Tupaia belangeri* (tupBel, Northern Treeshrew; v.1 Jun 2006), *Cavia porcellus* (cavPor, Guinea Pig; v.3 Mar 2008), *Sus scorfa* (susSco, Pig; v.10.2 Aug 2011), *Mus musculus* (musMus, Mouse; v.37 Apr 2007), *Rattus norvegicus* (rarNor, Rat; v.3.4 Dec 2004), *Spermophilus tridecemlineatus* (speTri, Squirrel; v.2 Nov 2011), *Ochotona princeps* (ochPri, Pika; v.2.0 Jun 2007), *Oryctolagus cuniculus* (oryCun, Rabbit; v.2 Nov 2009), *Ailuropoda melanoleuca* (ailMel, Panda; v.1 Jul 2009), *Canis lupus familiaris* (canFam, Dog; v.2.0 May 2006), *Felis catus* (felCat, Cat; Mar 2006), *Equus caballus* (equCab, Horse; v.2 Sep 2007), *Pteropus vampyrus* (pteVam, Megabat; v.1 Jul 2008), *Bos taurus* (bosTau, Cow; v.3.1 Nov 2009), *Tursiops truncates* (turTru, Dolphin; v.1 Jul 2008), *Choloepus hoffmanni* (choHof, Sloth; v.1 Sep 2008), *Loxodonta africana* (loxAfr, Elephant; v.3.0 Jul 2009).
(PDF)

Figure S6 **RCAN3-associated CpG island sequence conservation between mammals and in silico prediction of trancription factor binding sites.** Alignment of 18 mammalian genomic sequences corresponding to the CpG island associated with human *RCAN3* (based on UCSC reported CpG island). The different TFBS conserved among most of the organisms analysed are indicated with black boxes. Species and genome sequence versions used were as indicated in Figure S5 with the addition of *Erinaceus europaeus* (eriEur, Hedgehog; v.1 Jun 2006), *Pteropus vampyrus* (pteVam, Megabat; v.1 Jul 2008), *Nomascus leucogenys* (nomLeu, Gibbon; v.1.0 Jan 2010), and *Tupaia belangeri* (tupBel, Tree Shrew; v.1 Jun 2006). Grey intensity shade increases with sequence conservation (50, 70 and 90% of nucleotide identity). Numbers correspond to nucleotide coordinates referring to the first position of the *Homo sapiens* CpG island. Exon 1 spans positions 1 to 90; exon 2a, positions 521 to 741; and exon 2, positions 521 to 774.
(PDF)

Table S1 **Paralogous genes located in human chromosomes 1 (1p32-p36.3) and 6 (6p12-p21.2/q12-q22.1).**
(XLSX)

Table S2 **Previous and novel nomenclature for RCAN3 exons, mRNA forms and protein isoforms.**
(XLSX)
