# Supplementary figures and images for: The Vertebrate RCAN Gene Family: Novel Insights into Evolution, Structure and Regulation
Source: PLoS One. 2014 Jan 20;9(1):e85539. doi: 10.1371/journal.pone.0085539 (PMC3896409; doi:10.1371/journal.pone.0085539)

Figure S1

A

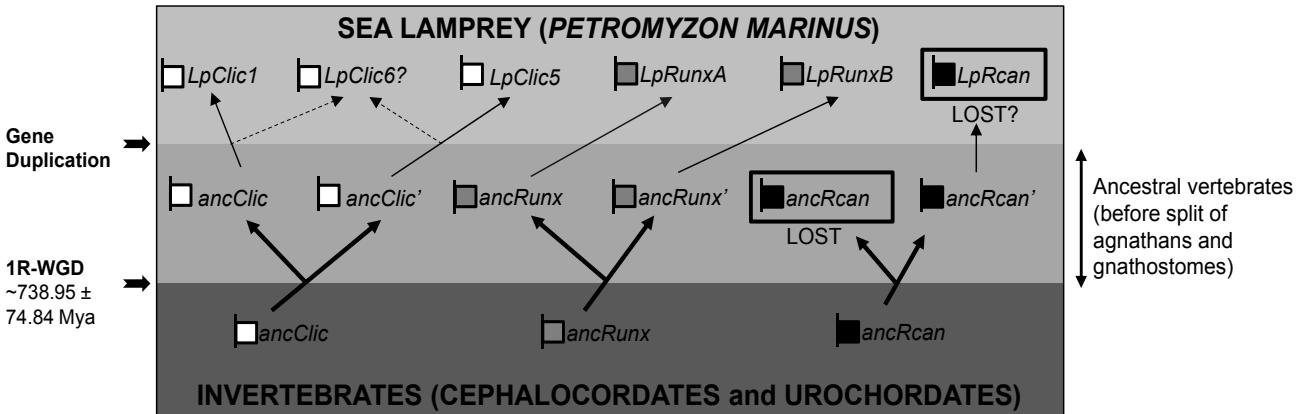

B

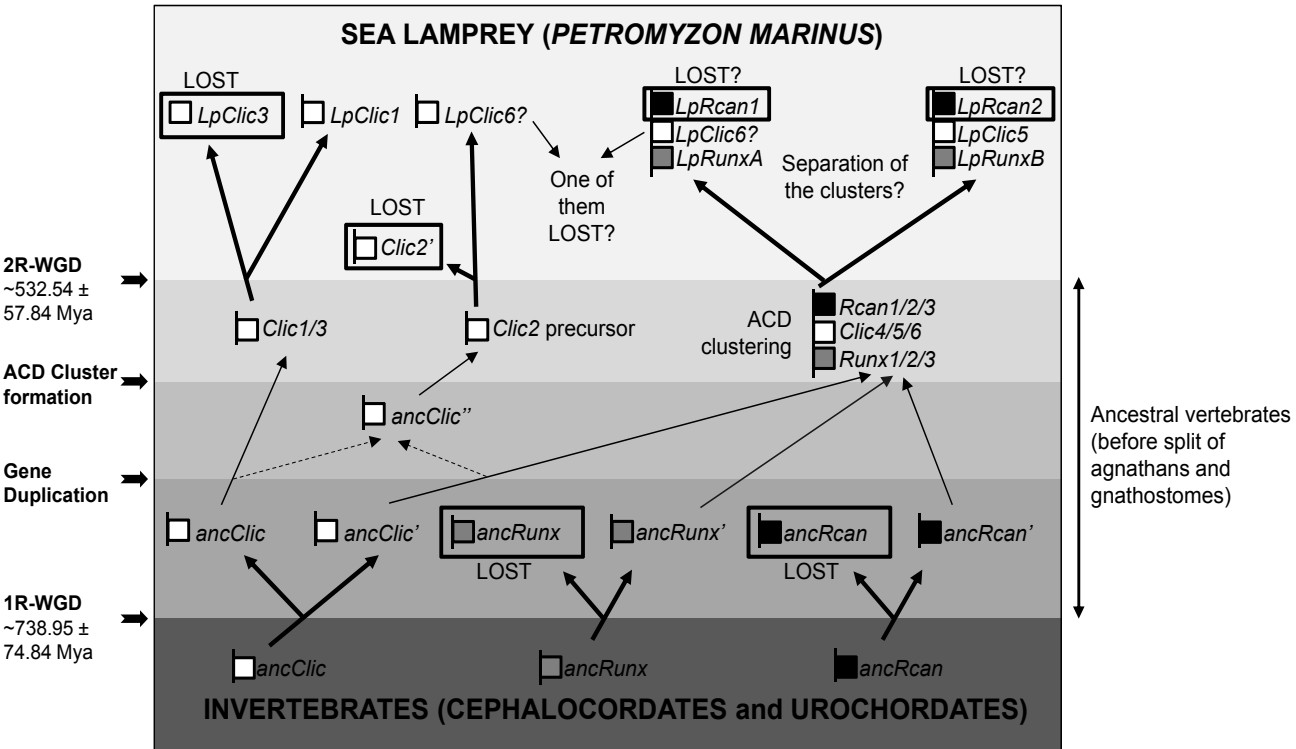

Supplement: Figure S1 — Alternative proposed scenarios for the evolution of Runx, Clic and Rcan genes in sea lamprey (Petromyzon marinus). Evolutionary hypothesis for Runx, Clic, Rcan genes in sea lamprey, considering that sea lamprey only underwent the first round of WGD (A) or that it also underwent the second round of WGD (B). Estimated times of 1R-WGD and 2R-WGD were obtained from Vienne et al. [58]. Thicker arrows indicate gene duplication events and the black-framed boxes correspond to gene losses. Abbreviations: anc, ancestral; agn, agnathans; gna, gnathostomes; Mya, Million Years Ago; WGD, Whole Genome Duplication. (PDF) [file pone.0085539.s001.pdf]

Figure S2

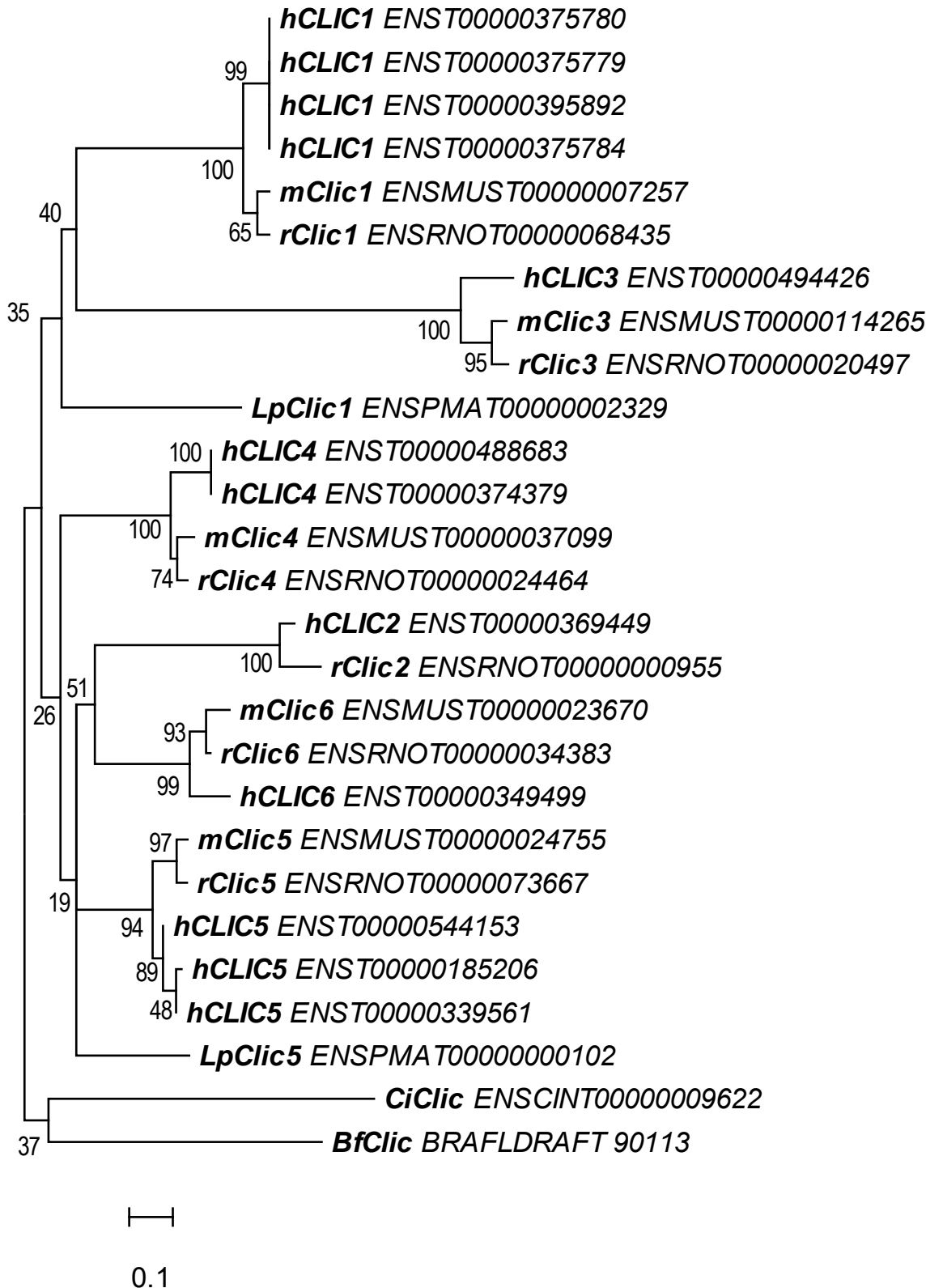

Supplement: Figure S2 — Phylogenetic analysis of CLIC transcripts. Coding DNA sequences (CDS) of the known functional Lamprey Clic (named LpClic1 and LpClic5) were retrieved from the Ensembl database [32] and used for subsequent phylogenetic analysis, as described in the Material and Methods section, together with all human, mouse, rat, sea squirt (Ciona intestinalis) and amphioxus (Branchiostoma floridae) CLIC CDS (hCLIC, mClic, rClic, CiClic and BfClic; respectively). Note that the Ensembl database contains three lamprey Clic genes: LpClic1, LpClic5 and LpClic6. Since the LpClic6 sequence is incomplete, it has been excluded from the analysis. The evolutionary tree obtained shown here relates LpClic1 with human and rodent CLIC1/3 and LpClic5 with human and rodent CLIC4/5/6. Ensembl names for each transcript and Uniprot reference for Branchiostoma floridae Clic are indicated). Numbers at the tree nodes correspond to bootstrap values. The scale bar in the bottom refers to the branch lengths and the number indicates substitutions per site. (PDF) [file pone.0085539.s002.pdf]

Figure S4

A

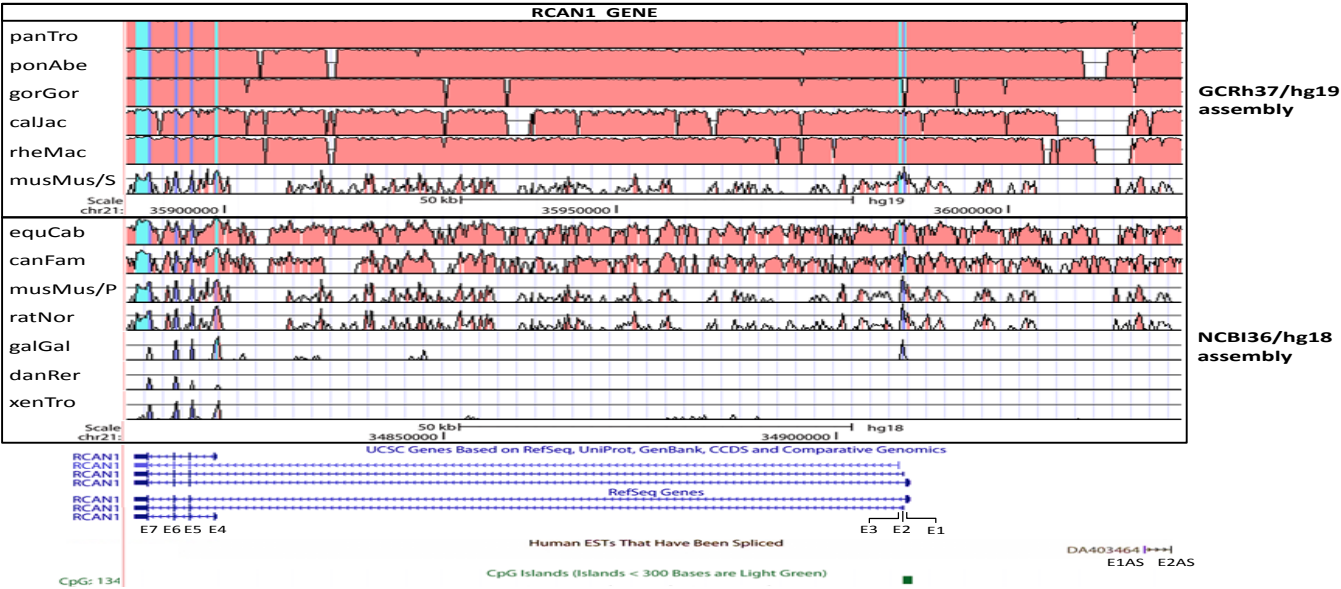

B

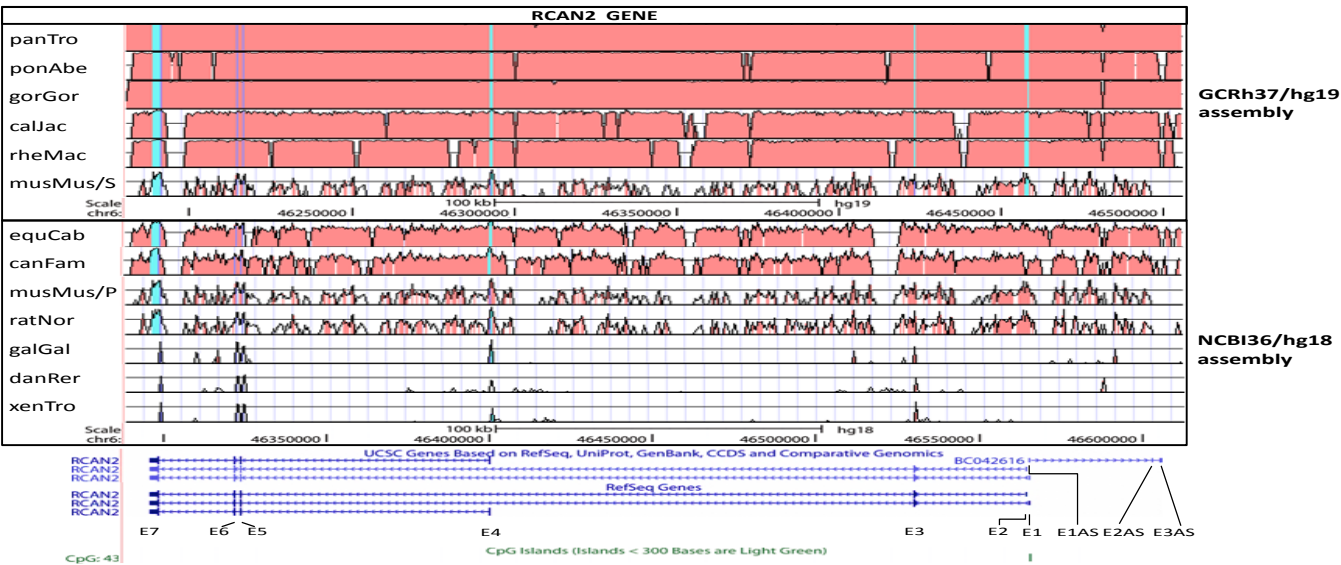

C

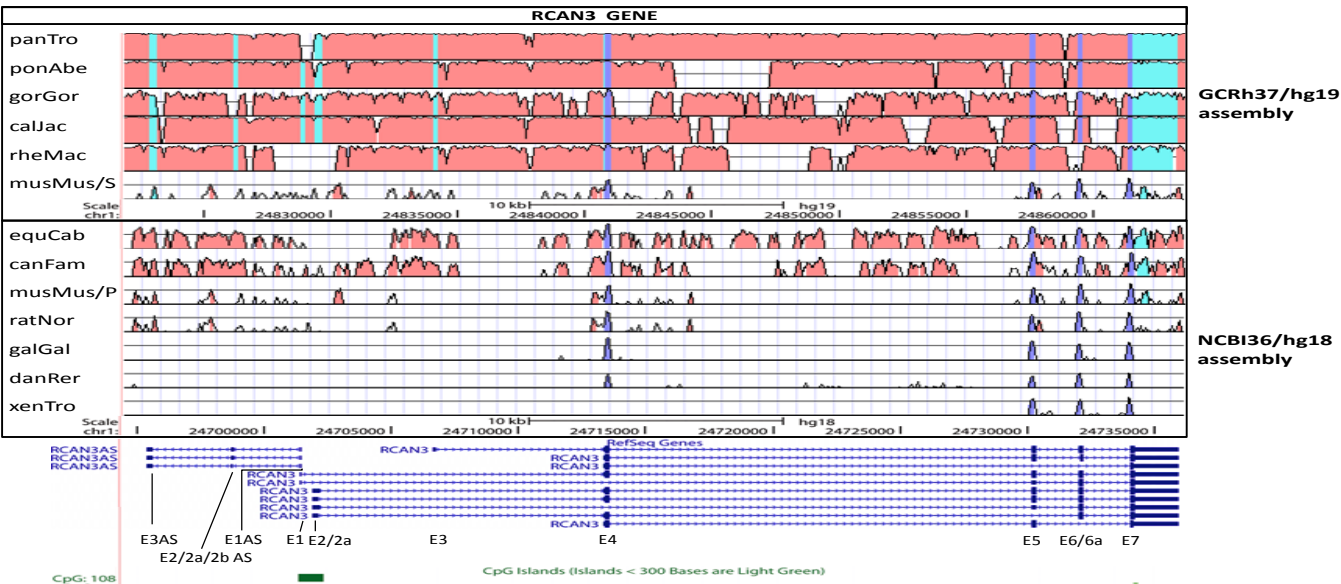

Supplement: Figure S4 — Comparative genomic sequence analysis of RCAN genes. Alignment plots of RCAN1 (A), RCAN2 (B) and RCAN3 (C) orthologs against human RCAN genes created by the VISTA tool of the UCSC browser [34], [52] using the Feb.2009-GRCh37/hg19 genome version for primates and mouse (SLAGAN alignment) and Mar.2006-NCBI36/hg18 for the other organisms. The vertebrate organisms used in the alignments against human sequences were: Pan troglodites (panTro), Pongo abelii (ponAbe), Gorilla gorilla (gorGor), Callithrix jacchus (calJac), Macaca mulatta (rheMac), Mus musculus (musMus), Equus caballus (equCab), Canis lupus familiaris (canFam), Rattus norvegicus (ratNor), Gallus gallus (galGal), Danio rerio (danRer), and Xenopus tropicalis (xenTro). The genome versions are indicated in the Material and Methods section. The chromosomal scale and base pair position guide are represented below the global comparative alignment in each assembly. All alignments are PROLAGAN alignments except when specified otherwise. In plots, darker blue indicates coding regions; lighter blue, untranslated regions (UTR) and pink, non-coding DNA sequence. 5′ UTR and 3′ UTR regions are conserved in primates and, to a lesser extent, in other mammals, although they are not always annotated. RCAN RefSeq and UCSC transcripts are shown below the alignment. Arrows in transcripts indicate the sense of gene transcription and, therefore, the order of the exons (darker blue boxes). UCSC registered CpG islands are indicated below in green. (A) Comparative genomic analysis of vertebrate RCAN1 genes relative to the human gene (NCBI Gene ID: 1827). The natural antisense transcript (NAT) DA403464 is registered as human UCSC EST. (B) Comparative genomic analysis of vertebrate RCAN2 genes relative to the human gene (NCBI Gene ID: 10231). BC042616 gene (RCAN2AS) is registered as UCSC gene. (C) Comparative analysis of the vertebrate RCAN3 genes relative to the human gene (NCBI Gene ID: 11123). RCAN3 transcripts and RCAN3AS NATs, accept [file pone.0085539.s004.pdf]

Figure S5

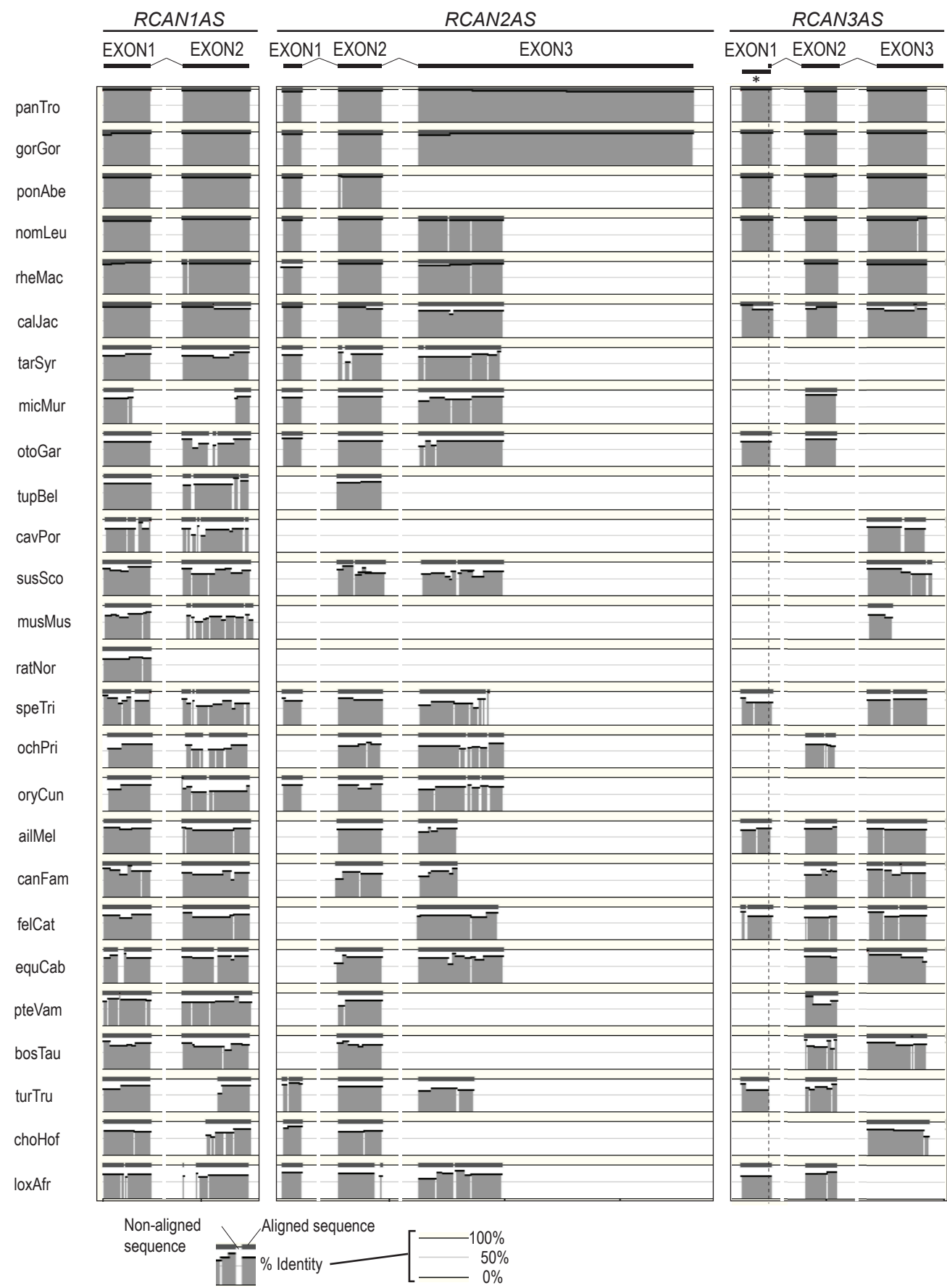

Supplement: Figure S5 — Multi-species alignment of human RCAN1, RCAN2 and RCAN3 natural antisense transcripts: RCAN1AS, RCAN2AS and RCAN3AS. Pip-type graph conservation profile of human RCAN1AS (DA403464 EST), RCAN2AS (BC042616 gene) and RCAN3AS (NCBI Gene ID: 100750325; [31]) in several eutherian mammals. Sequences were obtained using the genomic comparison tool of the Ensembl database [32] and alignments were generated and visualized by zPicture software [39]. Asterisk (*) indicates the region of exon 1 of RCAN3-1 transcript that was manually included to the alignment due to the impossibility of aligning sequences shorter than 19 nt (RCAN3AS exon 1 length is only 13 nt). Non-aligned regions correspond to gaps in the genome sequence. Species and genomes assemblies used for the analysis were: Homo sapiens (v.GRCh37.p7 Feb 2009), Pan troglodites (panTro, Chimpanzee; v.2.1.4 Feb 2011), Gorilla gorilla (gorGor, v.3.1 Dec 2009), Pongo abelii (ponAbe, Orangutan; v.2 Sep 2007), Nomascus leucogenys (nomLeu1.0, Gibbon; v. Jan 2010), Macaca mulatta (rheMac; v.1.0 Feb 2006), Callithrix jacchus (calJac, Marmoset; v.3.2.1 Jan 2010), Tarsius syrichta (tarSyr, Tarsier; v.1 Jul 2008), Microcebus murinus (micMur, Gray Mouse Lemur; v.1 Jun 2007), Otolemur garnettii (otoGar, Bushbaby; v.3 Mar 2011), Tupaia belangeri (tupBel, Northern Treeshrew; v.1 Jun 2006), Cavia porcellus (cavPor, Guinea Pig; v.3 Mar 2008), Sus scorfa (susSco, Pig; v.10.2 Aug 2011), Mus musculus (musMus, Mouse; v.37 Apr 2007), Rattus norvegicus (rarNor, Rat; v.3.4 Dec 2004), Spermophilus tridecemlineatus (speTri, Squirrel; v.2 Nov 2011), Ochotona princeps (ochPri, Pika; v.2.0 Jun 2007), Oryctolagus cuniculus (oryCun, Rabbit; v.2 Nov 2009), Ailuropoda melanoleuca (ailMel, Panda; v.1 Jul 2009), Canis lupus familiaris (canFam, Dog; v.2.0 May 2006), Felis catus (felCat, Cat; Mar 2006), Equus caballus (equCab, Horse; v.2 Sep 2007), Pteropus vampyrus (pteVam, Megabat; v.1 Jul 2008), Bos taurus (bosTau, Cow; v.3.1 Nov 2009), Tursiops truncates [file pone.0085539.s005.pdf]

Figure S6

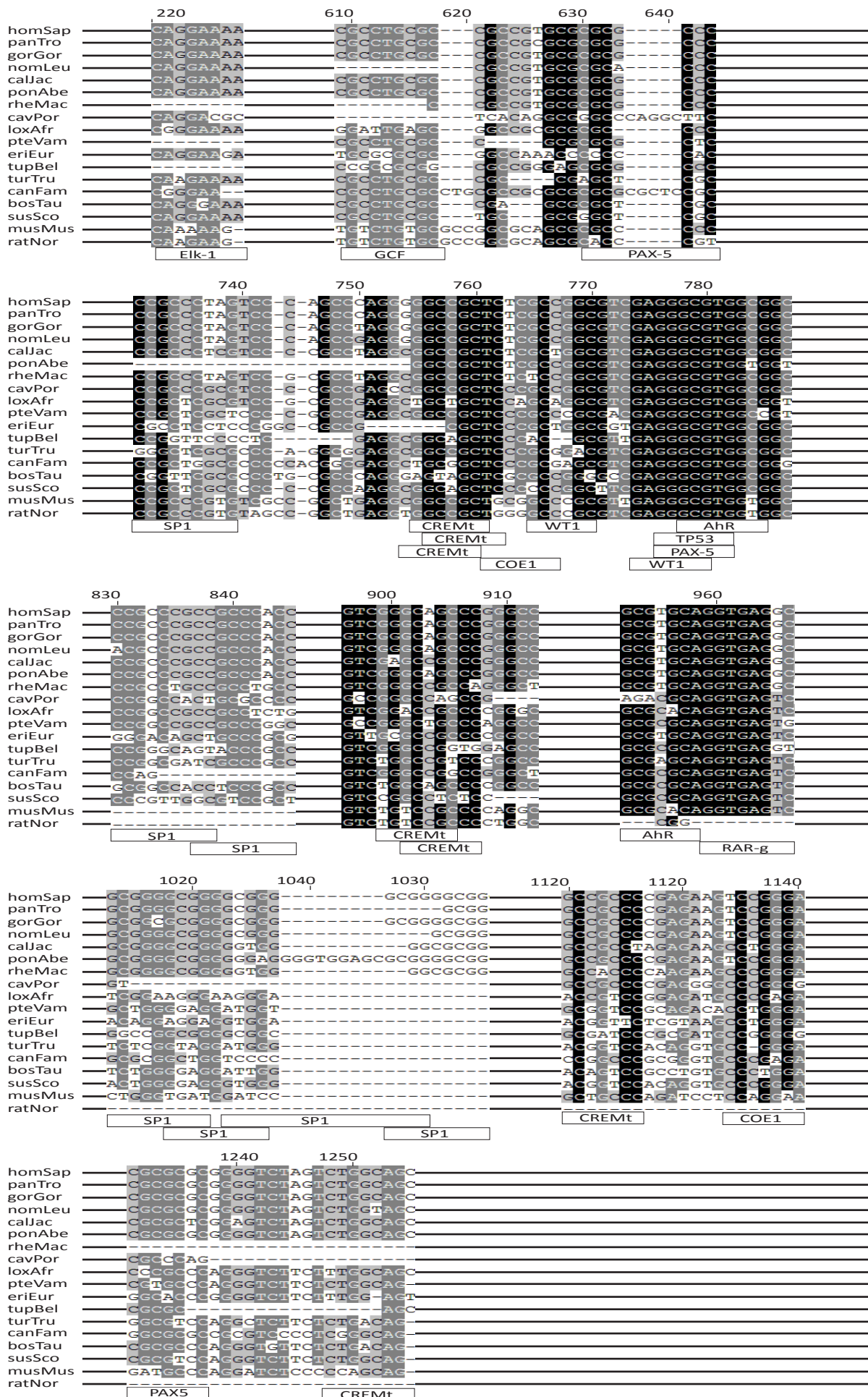

Supplement: Figure S6 — RCAN3-associated CpG island sequence conservation between mammals and in silico prediction of trancription factor binding sites. Alignment of 18 mammalian genomic sequences corresponding to the CpG island associated with human RCAN3 (based on UCSC reported CpG island). The different TFBS conserved among most of the organisms analysed are indicated with black boxes. Species and genome sequence versions used were as indicated in Figure S5 with the addition of Erinaceus europaeus (eriEur, Hedgehog; v.1 Jun 2006), Pteropus vampyrus (pteVam, Megabat; v.1 Jul 2008), Nomascus leucogenys (nomLeu, Gibbon; v.1.0 Jan 2010), and Tupaia belangeri (tupBel, Tree Shrew; v.1 Jun 2006). Grey intensity shade increases with sequence conservation (50, 70 and 90% of nucleotide identity). Numbers correspond to nucleotide coordinates referring to the first position of the Homo sapiens CpG island. Exon 1 spans positions 1 to 90; exon 2a, positions 521 to 741; and exon 2, positions 521 to 774. (PDF) [file pone.0085539.s006.pdf]
