# Supplementary material for: The Vertebrate RCAN Gene Family: Novel Insights into Evolution, Structure and Regulation
Source: PLoS One. 2014 Jan 20;9(1):e85539. doi: 10.1371/journal.pone.0085539 (PMC3896409; doi:10.1371/journal.pone.0085539)
Supplement: Figure S3 — Alignment of human RCAN proteins. (A) Schematic representation of RCAN proteins structure, indicating the coding exons. The last three exons (exon 5, 6 and 7, following the novel nomenclature proposed here for RCAN3) are common to all RCAN isoforms; and the variable exons result mainly from alternative transcription start site (TSS) usage. GSK3β (*) and MAPK, BMK1 or DYRK1A (†) phosphorylation sites within the FLISPP motif, important in RCAN regulation, are indicated. Additionally to these phosphorylation sites described to be common to all human RCANs, it has been recently characterized an additional site in RCAN3 (Ser 203) and RCAN1 (Ser 218) that are able to be phosphorylated in vivo by CK2α [15]. (B) Table indicates percentage of amino acid conservation between regions common to all human RCAN proteins (encoded by exon 5 to 7, according to the new proposed nomenclature). (C) Protein sequence alignment among the different protein isoforms encoded by human RCAN genes protein RefSeq acc. number: RCAN1-1, NP_004405.3 (252 aa); RCAN1-4, NP_981963.1 (197 aa); RCAN2-3, NP_005813.2 (197 aa); RCAN2-4, NP_001238902.1/NP_001238903.1 (243 aa); RCAN3-4, NP_038469.1/NP_001238906.1/NP_001238907.1/NP_001238908.1 (241 aa). All of them share exons 5 to 7, according to the new proposed nomenclature. Some conserved residues appear even in the protein region encoded by the first exon, which may be important for its functional activity and/or regulation. Grey intensity shade increases with sequence conservation (50, 80 or 100% of amino acid conservation). Numbers correspond to amino acid positions for each protein. (PDF) [file pone.0085539.s003.pdf]

Figure S3

A

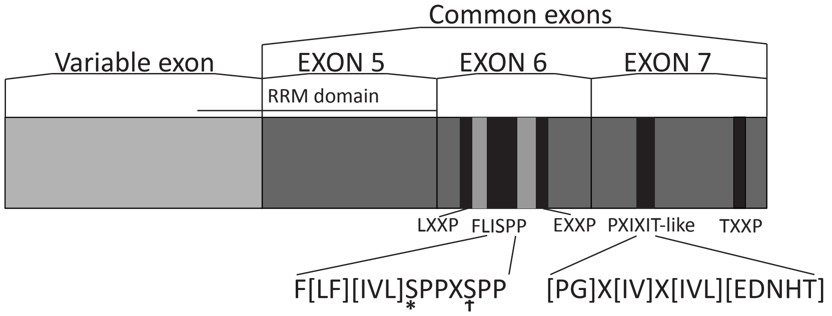

B

	hRCAN1-1	hRCAN1-4	hRCAN2-3	hRCAN2-4	hRCAN3
**hRCAN1-1**	100%	-	-	-	-
**hRCAN1-4**	100%	100%	-	-	-
**hRCAN2-3**	65%	65%	100%	-	-
**hRCAN2-4**	65%	65%	100%	100%	-
**hRCAN3**	60%	60%	68%	68%	100%

C

```
                          |        20         |         40         |        60         |
RCAN1-1    1 : MEDGVAGPQLGAAAEAAEAAEARARPGVTLRPFAPLSGAAEADEGGGDWSFIDCE--------------M :  56
RCAN1-4    1 : ---------------------------------------------------------------------M :   1
RCAN2-3    1 : ---------------------MRGESYFIGMRSPGQQGHVPEDGGLFLLCCIDRDWAVTRCFA--EEAFQ :  47
RCAN2-4    1 : ---------------------------------------------------------------------M :   1
RCAN3-4    1 : ---------------------------------MLRDTMKSWNDSQSDLCSTDQEEEEEMIFGENEDDLD :  37

                       80         |        100         |        120         |        140
RCAN1-1   57 : EEVDLQDLPSATIACHLDPRVFVDGLCRAKFESLFRTYDKDITFQYFKSFKRVRINFSNPFSAADARLQL : 126
RCAN1-4    2 : HFRNFNYSFSSLIACVANSDIFSESETRAKFESLFRTYDKDITFQYFKSFKRVRINFSNPFSAADARLQL :  71
RCAN2-3   48 : AITDFNDLPNSLFACNVHQSVFEGEESKEKFEGLFRTYDDCVTFQLFKSFRRVRINFSNPKSAARARIEL : 117
RCAN2-4    2 : PAPSMDCDVSTLVACVVDVEVFTNQEVKEKFEGLFRTYDDCVTFQLFKSFRRVRINFSNPKSAARARIEL :  71
RCAN3-4   38 : EMMDLSDLPTSLFACSVHEAVFEAREQKERFEALFTIYDDQVTFQLFKSFRRVRINFSKPEAAARARIEL : 107

                         |        160         |        180         |        200         |
RCAN1-1  127 : HKTEFLGKEMKLYFAQTLHIGSS----HLAPPNPDKQFLISPPASPPVGWKQVEDATPVINYDLLYAISK : 192
RCAN1-4   72 : HKTEFLGKEMKLYFAQTLHIGSS----HLAPPNPDKQFLISPPASPPVGWKQVEDATPVINYDLLYAISK : 137
RCAN2-3  118 : HETQFRGKKLKLYFAQVQTPETDGDKLHLAPPQPAKQFLISPPSSPPVGWQPINDATPVLNYDLLYAVAK : 187
RCAN2-4   72 : HETQFRGKKLKLYFAQVQTPETDGDKLHLAPPQPAKQFLISPPSSPPVGWQPINDATPVLNYDLLYAVAK : 141
RCAN3-4  108 : HETDFNGQKLKLYFAQVQMSGEVRDKSYLLPPQPVKQFLISPPASPPVGWKQSEDAMPVINYDLLCAVSK : 177

                        220         |        240         |        260         |
RCAN1-1  193 : LGPGEKYELHAATDTTPSVVVHVCESDQEKEEEEMERMRRPKPKIIQTRRPEYTPIHLS--------- : 252
RCAN1-4  138 : LGPGEKYELHAATDTTPSVVVHVCESDQEKEEEEMERMRRPKPKIIQTRRPEYTPIHLS--------- : 197
RCAN2-3  188 : LGPGEKYELHAGTESTPSVVVHVCDSDIEEEEDPKT----SPKPKIIQTRRPGLPPSVSN--------- : 243
RCAN2-4  142 : LGPGEKYELHAGTESTPSVVVHVCDSDIEEEEDPKT----SPKPKIIQTRRPGLPPSVSN--------- : 197
RCAN3-4  178 : LGPGEKYELHAGTESTPSVVVHVCESETEEEEETK-----NPKQKIAQTRRPDPPTAALNEPQTFDCAL : 241
```
